# Raman gas self-organizing into deep nano-trap lattice

**DOI:** 10.1038/ncomms12779

**Published:** 2016-09-28

**Authors:** M. Alharbi, A. Husakou, M. Chafer, B. Debord, F. Gérôme, F. Benabid

**Affiliations:** 1 GPPMM Group, XLIM Research Institute, CNRS UMR 7252, University of Limoges, Limoges 87410, France; 2 Physics Department, University of Bath, Bath BA2 7AY, UK; 3 Max Born Institute of Nonlinear Optics and Short Pulse Spectroscopy, Berlin 12489, Germany

## Abstract

Trapping or cooling molecules has rallied a long-standing effort for its impact in exploring new frontiers in physics and in finding new phase of matter for quantum technologies. Here we demonstrate a system for light-trapping molecules and stimulated Raman scattering based on optically self-nanostructured molecular hydrogen in hollow-core photonic crystal fibre. A lattice is formed by a periodic and ultra-deep potential caused by a spatially modulated Raman saturation, where Raman-active molecules are strongly localized in a one-dimensional array of nanometre-wide sections. Only these trapped molecules participate in stimulated Raman scattering, generating high-power forward and backward Stokes continuous-wave laser radiation in the Lamb–Dicke regime with sub-Doppler emission spectrum. The spectrum exhibits a central line with a sub-recoil linewidth as low as ∼14 kHz, more than five orders of magnitude narrower than conventional-Raman pressure-broadened linewidth, and sidebands comprising Mollow triplet, motional sidebands and four-wave mixing.

## Abstract (web summary)

Engineering the interaction between optical fields and gas-phase matters is important for spectroscopy and more general laser science and technology. Here, the authors demonstrate a method for light-trapping of molecular hydrogen in hollow-core photonic-crystal fibres, relying on Raman transition.

## Graphical abstract

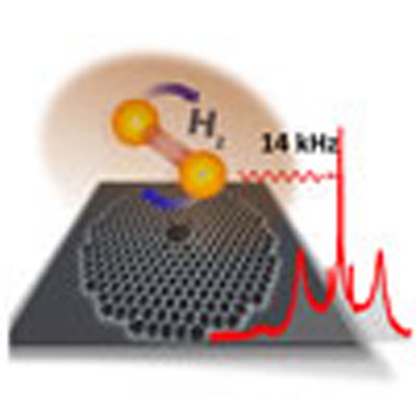

Engineering the interaction between optical fields and gas-phase matter is at the core of many key fields in modern physics, such as high-resolution spectroscopy, quantum optics, and laser science and technology. However, because atoms or molecules undergo random thermal motion and delocalization, their spectral lines are Doppler broadened, thus obscuring the observation of their quantum dynamics and destroying their coherence. As a result, probing and controlling these atoms/molecules coherently and at their single-quantum state has always been very challenging. Among the numerous laser-based interaction schemes to reach the sub-Doppler resolution, two broad approaches have emerged in the last three decades. The first one consists of cooling down the atoms and thus reducing their thermal motion. The success of this approach is exemplified in the advent of laser cooling techniques^1^,^2^,^3^ and Bose–Einstein condensate^4^,^5^. These techniques rely on the momentum decrease of the atom during the absorption–emission cycle of one of its electronic dipole transitions. The second approach stems from the emergence of nanotechnology and relies on spatially localizing atoms or molecules to a fraction of the optical wavelength, and thus hindering random delocalization by engineering micro- and nano-devices. Here the nano-structuring of solid-state materials into, for example, semiconductor loops^6^ or quantum dots^7^ creates artificial atoms or molecules that mimic the light absorption and emission from a single, non-moving atom. Both approaches enabled the observation with unparalleled details of several sub-Doppler spectral signatures from absorption and emission, with features such as sub-recoil linewidth^8^, resolved atomic translational motion, vacuum Rabi splitting^6^, Mollow triplets and resolved-sideband cooling^9^.

Achieving such a level of high-resolution spectroscopy or motion control with neutral and gas-phase molecules is highly desirable as they offer larger prospects than atoms in shedding light on phenomena such as violation of fundamental symmetries, long-range dipole interactions and chemical process dynamics. Today, because of the complexity of the molecular energy structure^10^, laser cooling of molecules proved to be extremely challenging as illustrated by the milli-Kelvin level of the lowest molecular temperature achieved with laser cooling of diatomic molecules^11^, and consequently alternative techniques^12^,^13^,^14^ are being explored. These techniques, however, are not reaching the level of spectral resolution or motion control that is exhibited with atoms. For example, with buffer gas cooling, which is a method of cooling molecules by mixing them with helium gas in a cryogenic cell, the lowest cooling temperature achieved was 400 mK (ref. 14) corresponding roughly to 7 MHz Doppler width. The other approach is based on Stark decelerating a supersonic molecular beam via an electrostatic field^12^ or optical fields^13^. Here the potentials formed by the interaction between an applied electric field and the dipole moment are used to slow and trap cold molecules. This technique has been extended to having two counter-propagating laser pulses to create a moving periodic potential to trap the molecules^15^. At the current level of this technique the Doppler broadening, which could be achieved (even for stopped molecules) is on the scale of 50–100 MHz. This situation calls for exploring alternative routes both conceptually and technologically to bring the molecular control and probing to a comparable level to that achieved with atoms using laser cooling.

Here we report on a scheme that adds an alternative route to what was proposed before in the control of molecules. This route outstands by the nature of laser-gas coupling, which relies on Raman transition instead of the commonly used dipole transition. The technique enables a light scattering with sub-recoil spectral resolution, a spatial confinement that is well below the wavelength, a rich sub-Doppler dynamics, and high-power lasing capabilities. All these features are demonstrated in a simple experimental set-up. The experimental set-up consists of hydrogen molecules in hollow-core photonic crystal fibre (HC-PCF)^16^ being excited with a powerful continuous-wave laser. This extremely simple configuration leads to rich and multifarious physical dynamics. In particular, it enabled trapping of room-temperature high-pressure Raman-active molecules in a self-assembled optical lattice whose potential depth of ∼55 THz allows the velocity capture of ∼1,800 m s^–1^, and whose spatial structure enables to localize molecules in periodic sections not wider than 100 nm. The molecules create their own optical lattice by generating via stimulated Raman scattering (SRS) forward and backward Stokes waves from quantum noise and to form a standing wave along a section of the fibre. In turn, this Stokes optical lattice is sufficiently strong to bring the molecules into a high-energy saturated Raman state over most of its length and to leave the molecules in their ground quantum state (that is, Raman active) only over nanometre-wide sections centred at the Stokes standing wave nodes (that is, Lamb–Dicke regime). At each of these nanometre-wide sections, the Raman-active molecules are tightly trapped by a deep trapping well of a periodic potential. The depth of this trapping potential lattice is set by the Raman transition frequency, and the spatial structure of its nanometre wells is fixed by the molecule population Raman saturation. This potential feature and the quantum noise origin of its optical lattice differ from the previously known optical-lattice localization.

The outcome is an optically self-nanostructured gas-phase material (OS-GPM) that consists of several centimetres long one-dimensional array of ∼100,000 nano-traps containing nano-localized Raman-active molecules that scatter light in the Lamb–Dicke regime. We observed a generation of continuous-wave Stokes laser that combines a power level of up to 50 W and a sub-recoil linewidth down to ∼14 kHz. The linewidth is over four orders of magnitude narrower than the Doppler linewidth. The Stokes emission spectrum has a sub-Doppler structure that shows resolved sidebands due to Rabi splitting, trapped molecule translational motion and four-wave-mixing (FWM) inter-sidebands. A further feature that results from SRS generation is that the formed potential is an accelerating potential imparting an overall drag to the molecules. This is observable with the naked eye via an IR viewer. Also, akin to highly localized electrons in superlattice semiconductors driven by a static electric field or electrons in an insulator^17^,^18^, the trapped molecules remain strongly localized inside their deep wells even in the presence of lattice motion or by pressure collisions. Below we organize the article by starting with a description of the experimental arrangement, followed by a theoretical background explaining the unusual results and finish with a detailed experimentally backed description of OS-GPM microscopic and macroscopic dynamics.

## Results

### Experimental configuration for Stokes generation

**Figure 1:**
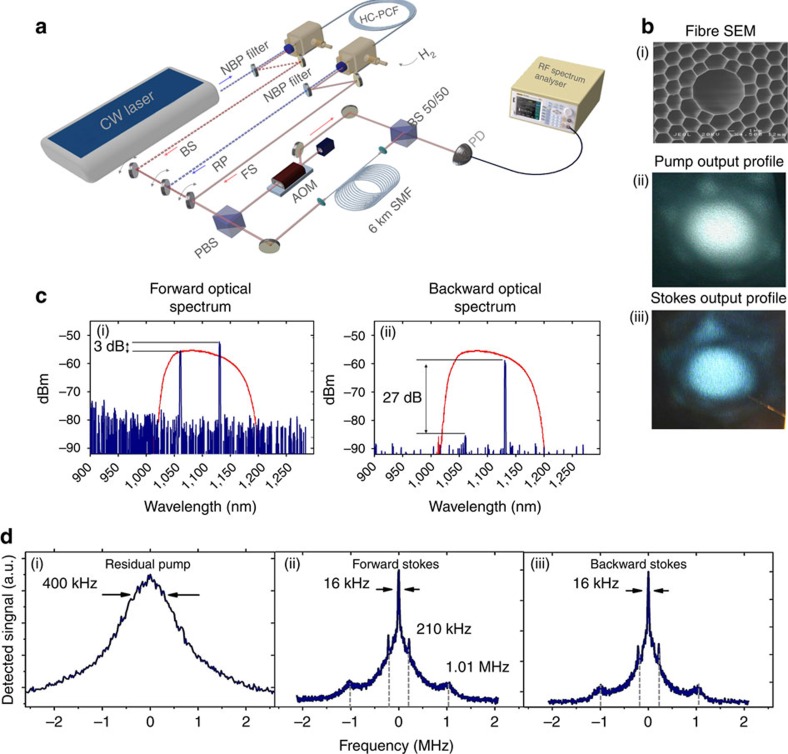
Stokes generation and linewidth measurement. (**a**) Schematic of the experimental set-up consisting of a high-power CW laser beam being coupled to a purposely designed hydrogen-filled HC-PCF so to only allow Stokes light generation from the first rotationally excited state in hydrogen gas. The light output from both ends of the fibre is monitored using an optical spectrum analyser to measure its spectral content, and a self-heterodyne unbalanced interferometer to measure the linewidth of each spectral component. AOM, acousto-optic modulator; BS, beam splitter; NBP filter, narrow bandpass filter; PBS, polarization beam splitter; PD, photodetector; RP, residual pump; SMF, single mode fibre. (**b**) Fibre scanning electron micrograph (i), laser beam profile of the transmitted pump (ii) and the generated forward Stokes (iii). (**c**) Optical spectra of the fibre transmission (red curve) and SRS spectra (blue curve) generated in the forward direction (i) and backward direction (ii). (**d**) Self-heterodyne radio-frequency (RF) spectra showing the linewidth of the transmitted residual pump (i), forward Stokes (ii) and backward Stokes (iii) for a pump power of 29 W and gas pressure of 20 bar.

Figure 1a schematically shows the experimental set-up (see Supplementary Note 1 for more details). A 20 m long home-made photonic bandgap guiding HC-PCF (top of Fig. 1b), filled with molecular hydrogen^19^ at uniform pressure in the range of 20–50 bar, is pumped with a randomly polarized 1,061 nm wavelength Yb-fibre continuous-wave (CW) laser. In such a system SRS occurs, where the incoming input laser photons transform into lower-energy (longer-wavelength) Stokes photons by transferring their energy difference to molecular rotational and/or vibrational transitions. The narrow transmission window of the HC-PCF (red curves of Fig. 1c) enables us to favourably excite only the first-order Stokes of the rotational transition *S*_00_(1) of H_2_ with a frequency shift of *ω*_R_∼2*π* × 17.8 THz (ref. 20). The generation of the second-order Stokes and anti-Stokes are inhibited by higher fibre transmission loss at the corresponding wavelengths. In a similar way, the vibrational SRS is suppressed because the Stokes frequency lies outside the fibre's transmission band^20^.

The blue curves in Fig. 1c show the optical spectra of the transmitted single mode (Fig. 1b) forward and backward beams for pump power of 29 W (that is, ∼17.5 W of coupled power) and hydrogen pressure of 20 bar. The forward optical spectrum shows two spectral lines; one at 1,061 nm from the pump laser (P) and a second at 1,131.4 nm from forward first-order Stokes (FS). The backward spectrum is composed mainly of the backward Stokes (BS) line, with residual light from the pump, which is back reflected off the fibre input end. Figure 1d shows radio-frequency spectra of typical linewidth traces over ±2 MHz spectral span of P, FS and BS. The linewidth of the fibre-transmitted pump was measured to be ∼ 400 kHz, consistent with the manufacturer's specifications. This linewidth value and spectral profile remain unchanged when working parameters are varied (Supplementary Note 2 and Supplementary Fig. 1).

The FS and BS linewidth traces, however, not only depend on the coupled pump power and the gas pressure (see below) but also markedly contrast with what one would expect from the conventional physical picture of SRS process, which predicts a pressure-broaden Stokes linewidth of around 2 GHz for a gas pressure of 30 bar (ref. 21). The present emitted Stokes lines stand out with a linewidth that is over five orders of magnitude narrower than the pressure broadened line. Here the measured linewidth of Δ*v*=16 kHz for both FS and BS are well below the Doppler-limit linewidth of 153 and 300 MHz for H_2_ rotational Raman transition in forward and background configurations, respectively^22^. Most remarkably, the measured linewidth is below the hydrogen molecule recoil frequency *v*_recoil_=*πħ*/(*mλ*_S_^ 2^) of ∼78 kHz. This means that not only collisional broadening is suppressed but also the broadening is below the limit caused be the molecular recoil on a photon emission. Furthermore, FS and BS linewidth spectra show two sidebands with a frequency shift of 210±15 kHz, and 1.01 MHz, respectively. These spectral characteristics are usually signatures of strongly trapped atoms in the Lamb–Dicke regime^23^. In our case, this Lamb–Dicke regime results from an original dynamic, which we outline below.

### Theoretical background

The present laser-gas interaction is modelled by treating the Raman medium as a two-level system
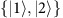
24 (see Supplementary Note 3 for a detailed account of the model and the numerical simulations). Here 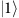 and 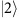 represent, respectively, the ground and excited states of the molecular rotation, and whose frequency difference *ω*_R_ corresponds to the molecular rotation resonance frequency. Under the presence of a pump and Stokes fields *E*_p_ and *E*_S_, the effective Hamiltonian of the system is 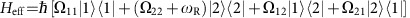. Here 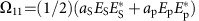 and 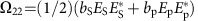 are the respective frequency shifts of 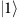
and 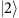 caused by the Stark effect. The strength of this SRS process is defined by the two-photon Rabi frequency 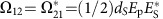, set by the mixing of the pump field with that of the Stokes. The quantities 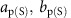 and *d*_S_ are the coupling coefficients related to the second-order dipole moments between the different states involved^25^. In our case, the Stokes field is amplified from the quantum noise, and is generated in both forward and backward directions to form a standing wave. The presence of this Stokes lattice is the first key feature to understand the molecular dynamics and the observed line narrowing. Indeed, the Stokes lattice imparts spatial modulation to Ω_12_, with a period of half the Stokes wavelength, *λ*_S_/2 as follows:


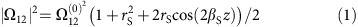


Here 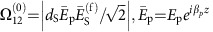, 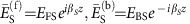 and 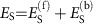. Quantity *β*_p(S)_ is the propagation constant for the pump (Stokes), and *r*_S_, defined by 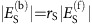, is the local ratio between the forward and backward components of the pump or Stokes field. The superscripts (f) and (b) stand for forward and backward. We ignored the backward pump as it is too small to have meaningful effect.

In turn, the two-Rabi frequency modulation induces a periodic distribution along *z* of the population difference *D*, Raman gain *g*_R_ and the expectation value 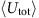
of the potential energy as shown below:


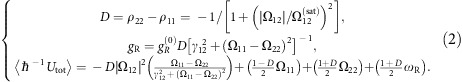


Here *ρ*_11_ and *ρ*_22_ are the diagonal elements of the two-level Raman system density matrix *ρ*, and 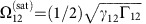 being the saturation two-photon Rabi frequency. The two quantities *γ*_12_∼2*π* × (*p*_g_/20) × 1.14 GHz (ref. 26) and Γ_12_∼2*π* × (*p*_g_/20) × 20 kHz (ref. 27) are Raman dephasing rate and population decay rate, respectively. We assume the system to be closed, meaning that *ρ*_11_+*ρ*_22_=1, *ρ*_11_=(1−*D*)/2 and *ρ*_22_=(1+*D*)/2. Finally, 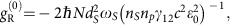 with *N*, *n*_S_, *n*_p_, *c* and *ɛ*_0_ are the molecular density, the Stokes and pump refractive indices, the vacuum light speed and the vacuum permittivity, respectively. In the expression of the potential energy, deduced from the trace formula 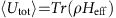, we can identify four terms. The first three terms are the Raman transition analogue of the a.c. Stark effect in a single-photon dipole transition. The first one corresponds to the energy change due to the SRS two-photon interaction between the two Stark-shifted states. The second and third are the energies associated with the Stark effect on the ground and excited states, respectively. Finally, the fourth term corresponds to the energy of the molecular rotation, and has no analogue in conventional dipole optical lattices.

**Figure 2:**
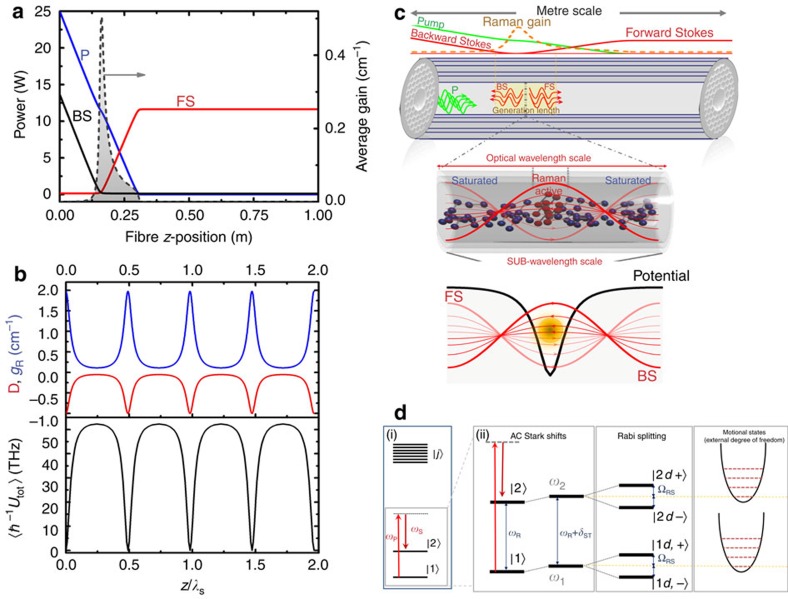
Self-optically nano-structured Raman-active molecules. (**a**) Numerical simulation of macroscopic power *z*-distribution along the fibre of the pump (blue curve), FS (red curve) and BS (black curve). The grey dashed and filled curve is the average Raman gain (in unit of cm^−1^). (**b**) Microscopic *z*-distribution (normalized to the Stokes wavelength) of the Raman gain (blue curve), the normalized population difference (red curve) and the expectation value of the potential (black curve). (**c**) Schematics of OS-GPM dynamics and scale from the metre scale of the hydrogen-filled fibre (top), to a single Stokes-wavelength scale (middle) to then nanometre scale (bottom). The power evolution along the fibre length of the pump, forward Stokes and backward Stokes is represented by a green curve and letter P, red curves and the letters FS and BS, respectively. The Raman gain distribution along the fibre is shown in brown dashed curve. The red spheres represent the Raman active molecules and the blue ones represent the Raman saturated molecules. (**d**) Schematics of the energy levels of the molecules inside the OS-GPM both for the internal degree of freedom (here hydrogen molecule rotation) (i), their Stark shift and Rabi splitting along with the external degree of freedom (translational motion of the molecules) (ii).

Examination of equation (2) indicates that both the Raman gain and the potential are directly dependent on the population difference, their spatial ordering can be markedly shaped for extreme values of *D*. Indeed, in the regime when *D*≈−1 the population is dominantly in the ground state, the Raman gain is at its maximum and corresponds to the most common SRS configuration, with the system energy being primarily determined by the Raman interaction and the stark shift difference between the two states. On the other hand, when *D*≈0, corresponding to the saturation of the SRS process, the Raman medium no longer scatters (that is, *g*_R_=0). In this regime the system is effectively transformed to that of an uncoupled two-level system with the ground and excited state being shifted via Stark shift, and the potential energy being dominated by the molecular rotation when *ω*_R_>>Ω_22_. Furthermore, in the presence of high level of field intensity (that is, 
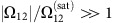
), which can be achieved even at very low power levels thanks to the small fibre core and large interaction length, the spatial modulation of the population induced by the presence of the Stokes lattice can be a mechanism to spatially alternate between the two above extreme regimes. The resulting potential lattice strongly differs from those used in existing techniques in confining atoms or molecules. First, its dominant energy term is not provided by the Stark effect like in conventional molecular or atomic localization experiments, but by the Raman rotation frequency. Second, its longitudinal profile, along with that of *D*, *g*_R_ is not that of the cosine shape of the conventional optical dipole lattices, and can achieve wells that orders of magnitude narrower than the optical wavelength. Figure 2 illustrates how such a spatial distribution in *D*, *g*_R_ and 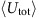 is self-generated from conventional SRS generation scheme, and captures the underlying macroscopic and microscopic dynamics, via numerical calculations for a representative case of *P*_in_=25 W and *p*_g_=20 bar (Fig. 2a,b), as well as schematically (Fig. 2c,d).

### Molecular macroscopic and microscopic structure

Figure 2a shows the numerically calculated power distribution along the fibre length of the pump (P), FS, BS and the Raman average gain 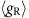, respectively. 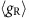
has an average value of 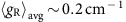. At this macroscopic scale, the evolution of P, FS, BS and 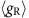 with the fibre length is typical for the conventional picture of SRS process^28^: the pump power is gradually depleted, and at ∼15 cm from the fibre input a quick rise of FS and BS takes place within the Raman generation length 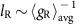. Outside this region, the Raman gain drops to almost zero. This macroscopic dynamic is also schematically illustrated in the top of Fig. 2c.

Within the Raman generation length, the system shows a microscopic sub-structure that contrasts with prior work on SRS. Here the three quantities *D*, *g*_R_ and 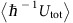 exhibit a spatial modulation, which has a period of *λ*_S_/2 and alternates between a nanometre-wide Raman-active region (RAR; that is, *D*∼−1) and a Raman-saturated region (that is, *D*∼0) (Fig. 2b). The RAR corresponds to maximum gain (see blue curve of Fig. 2b), and the molecules are in the ground state (see red curve of Fig. 2b). This is explained by the fact that the centre of RAR is located at the anti-node of the Stokes standing wave, where the two-photon Rabi frequency is nil. In Raman-saturated region, the Raman gain is close to zero due to the strong two-photon Rabi frequency, which drives the Raman molecules to saturation, and to SRS suppression. The net result is that SRS occurs only from the nano-wide RAR regions. For the representative case, the width of these regions was found to be ∼38 nm, which is much narrower than the Stokes wavelength. The width narrowing is directly proportional to the Raman saturation level, as illustrated by the analytical expression of the RAR width 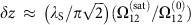 for the case of *r*_S_=1. Furthermore, Fig. 2b (black curve) shows that the RAR is also the site of an extremely deep trapping well with a depth along the *z*-direction, 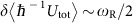 of more than 55 THz corresponding thus to the staggering figures of velocity capture of 1,800 m s^–1^ and effective temperature of ∼425 K. This large velocity capture is the second key feature of the physical mechanism: both the trapping of the molecules and the spatial modulation of saturation lead to the combination of drastic reduction of the linewidth through the Lamb–Dicke mechanism, and strong SRS process. This 55 THz potential depth is several orders of magnitude larger than the few-MHz potential depth of typical optical atom traps. It also leads to the approximately fourfold increase of the molecules density in the nano-sections as estimated by Boltzmann distribution, partly explaining the strong quantum conversion to the Stokes (Supplementary Note 10). This OS-GPM is schematically illustrated in the bottom of Fig. 2c where the scattering molecules (red coloured) are ‘sandwiched' between saturated molecules (blue coloured) within the nano-wide section and are kept there with the trapping potential forces.

### Line narrowing and sideband generation dynamics

**Figure 3:**
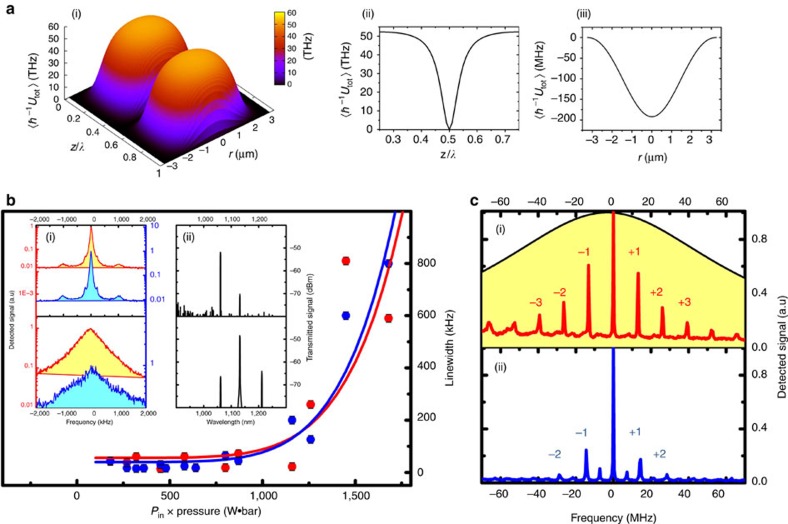
Measured Stokes-linewidth spectral structure. (**a**) Calculated microscopic three-dimensional profile of the potential (i). Potential longitudinal profile (ii) and transverse profile (iii) in the vicinity of an anti-node (that is, at *z*=*n λ*_S_/2, with *n* being an integer). (**b**) FS (red colour) and BS (blue colour) measured linewidth evolution with *P*_in_ × *p*_g_. The points are experimental data and the solid lines are fitted curves to the experimental points. Inset: FS (yellow filled) and BS (blue filled) linewidth spectral traces over 4 MHz span (i) and transmitted optical spectra (ii) for *P*_in_=9 W and *p*_g_=20 bar (top), and for *P*_in_=29 W, and *p*_g_=50 bar (bottom). (**c**) FS (red curve) and BS (blue curve) spectrum over 150 MHz span. The BS Doppler-limited linewidth (yellow-filled curve) is shown for comparison.

This picture of deeply trapped scattering molecules in a potential depicted in Fig. 3a, which is further corroborated below, explains the observed combination of a strong conversion to the Stokes (Fig. 1c), the narrow linewidth (Fig. 1d) and the resolved sidebands (Fig. 3b,c).

Indeed, with such potential, the expected Lamb–Dicke^23^ narrowing of the spectrum is given by the ratio 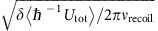 (ref. 29) of ∼1 × 10^4^, which is consistent with the ratio of ∼10^4^ between the measured FS minimum linewidth of ∼14 kHz and its corresponding Doppler-limited of 153 MHz (Fig. 2d). The strong Lamb–Dicke line narrowing is investigated as a function of the intensity-pressure product as shown in Fig. 3b (see Supplementary Notes 4 and 5, and Supplementary Figs 2–5 and 8–11 for larger data set). Each point in Fig. 3b represents the average of several linewidth measurements from the recorded radio-frequency spectra and their nonlinear fit (see Supplementary Note 4, and Supplementary Figs 6 and 7 for the details on the linewidth measurements). The figure shows two regimes in the evolution of FS and BS linewidths with pump power and gas pressure. Within our explored input power and gas pressure ranges corresponding to *P*_in_ × *p*_g_≲1,000 W bar, the linewidth varies little and found to be in the range between 14 and 72 kHz with an uncertainty of ±10 kHz. All the measured linewidths in this pump power and gas pressure are below the recoil limit. The small linewidth variation in this range is explained by the fact that the potential depth 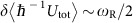 is mostly determined by the intensity- and pressure-independent Raman transition frequency, with the Stark and Rabi frequencies being several orders of magnitude smaller. Above 1,000 W bar, the Stokes linewidth broadens and exhibits a ‘triangular' profile (bottom of Fig. 3b(i)). Its onset coincides with the generation of the second-order Stokes (see bottom of Fig. 3b(ii)), which alters the OS-GPM structure, thus affecting the trapping dynamics described above. We note, however, that it is possible to operate in the above-mentioned Lamb–Dicke regime at larger input power levels by scaling the gas pressure and fibre length (Supplementary Note 7, and Supplementary Figs 14 and 15).

Within this regime of sub-wavelength localization and strong driving fields, the molecular internal energy structure is altered. Figure 2d illustrates the expected changes to energy levels. Under a strong pump, the unperturbed energy levels of the conventional two-level system (Fig. 2d(i)) are a.c.-Stark shifted and Rabi split (Fig. 2d(ii)). Hence, the system is now better described by a four-level system dressed states^1^ 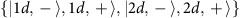 (Fig. 2d(ii)), and the scattering spectrum would exhibit Mollow triplet spaced by the two-photon Rabi frequency. This is experimentally corroborated in Fig. 3c, which shows FS and BS spectral structure over a larger span of 150 MHz for the case of *P*_in_=16 W and *p*_g_=50 bar. In addition to the sub-recoil central peak, the spectra exhibit several strong equally spaced sidebands from the central peak (labelled ±1, ±2, … in Fig. 3c). The corresponding FS Doppler-limited linewidth is shown with yellow filled-curve to evidence for the sub-Doppler regime of the generated spectrum (Fig. 3c(i)). The measured FS sideband spacing of 13.2 MHz is in good agreement with the numerically calculated effective two-photon Rabi frequency Ω_RS_=10.2 MHz at the nano-traps (Supplementary Note 8). The agreement between theory and experiment is also confirmed in the fact that Ω_RS_ varies little with *P*_in_. Consequently, the first two lines (that is, labelled ±1) form, together with the central line, the Mollow triplet. The higher-order lines (labelled ±2, ±3, …) are the results of inter-sidebands FWM that occurs via optical nonlinearity^30^.

Because the molecules are highly spatially localized, each dressed state of the molecular rotation should exhibit quantized translational-motion states represented in Fig. 2d(ii). Using the harmonic oscillator approximation, the present potential three-dimensional profile for the representative case of *P*_in_=25 W and *p*_g_=20 bar (Fig. 3a(i)) indicates two types of motional states: transverse and longitudinal. The transverse trapping with a well depth of ∼200 MHz (Fig. 3a(iii)) corresponding to transverse oscillations at a frequency 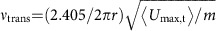 of ∼300 kHz. Here *U*_max,t_ is the potential local maximum along the transverse direction. The observed ±210 kHz sidebands shown in Fig. 1d are consistent with the transverse motional frequency *v*_trans_, which was found to be in the range of 200–400 kHz range for different pump powers and gas pressures. Furthermore, their narrower width relative to central peak is indicative of Lamb–Dicke narrowing in the transverse direction.

Figure 2d also illustrates a longitudinal trapping provided by a well-depth 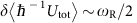 of more than 55 THz (Fig. 3a(ii)) and corresponding to a longitudinal motion with a frequency 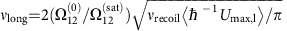, and which value spans from ∼3 to ∼17 GHz in the *P*_in_ × *p*_g_ range explored. This was outside our self-heterodyne detection range. Their existence was demonstrated by measuring the FS spectrum, for a pump power of 20 W and a pressure of 15 bar, by two means (Supplementary Note 6 for the full details and Supplementary Figs 12 and 13 for the spectra). The first one is achieved by recording the spatial profile of the FS diffracted beam, and the second with an optical spectrum analyser. Both measurements show a spectrum with three distinct peaks and with frequency spacing in the range of 10–16 GHz. Given the measurement uncertainty of our set-ups, which is ∼10 GHz, this is a very good agreement with the theoretical predictions. Furthermore, one of the spectra clearly shows an asymmetry in power between the sidebands, which is indicative of thermal distribution in a quantized harmonic oscillator (Supplementary Fig. 12). Follow-up work with better resolution is, however, necessary to explore the fine structure of these sidebands, but also to provide further insight into the dynamics of the molecules within the nano-traps and to explore whether this configuration leads to resolved sideband molecular cooling^31^.

### Lattice in motion

The BS spectrum (Fig. 3c(ii)) exhibits the same Mollow sidebands and their higher orders as FS. However, the spacing between the BS Rabi sidebands is 14.4 MHz, which is 1.2 MHz higher than for FS. This difference results from a Doppler shift between FS and BS, which is associated with drift velocity of the OS-GPM along *z*-direction of 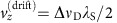 of 0.7 m s^–1^. As a side note, this frequency shift between FS and BS sidebands and their coexistence in the gain region leads to the FWM with the central peak, resulting in the sideband at roughly 1.1 MHz, which is indeed visible in Fig. 3b(i).

**Figure 4:**
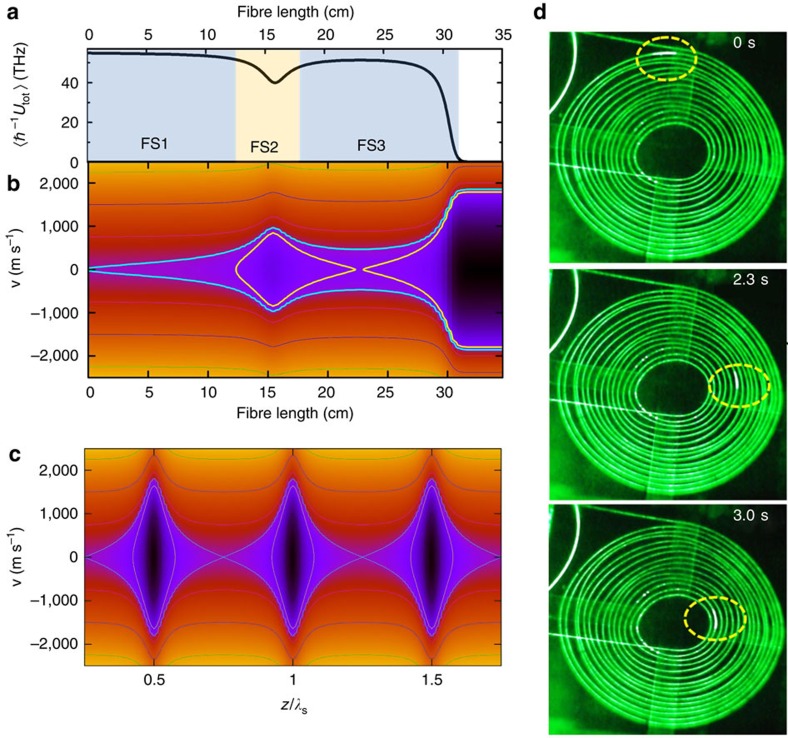
Molecular acceleration and macroscopic dynamics. Macroscopic *z*-distribution over the first 35 cm HC-PCF of the Hamiltonian (**a**) and the phase-space diagram (**b**). FS1, FS2 and FS3 represent the fibre sections where molecules experience different acceleration regimes. (**c**) Zoomed-in phase-space diagram over ∼2*λ*_S_ wide section from FS2. (**d**) Selected frames from the video (Supplementary Note 11 and Supplementary Movie 1) showing the moving scatter (dashed–circled sections) along the spirally set fibre. The frame corresponding time sequence is shown on the right top.

The reason for the drift is given in Fig. 4a, which presents the macroscopic average potential 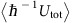 *z*-distribution over the first 35 cm fibre length. Over this length, the potential shows a significant gradient, and thus the molecules are under acceleration by virtue of the optical force 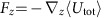. This potential gradient is a direct result of the pump depletion during SRS process and the Stokes generation (Fig. 2a). This also explains the parabolic profile of the average potential at the gain region (labelled FS2 in Fig. 4a). Within this non-moving FS2 region, where the OS-GPM opto-molecular lattice takes place, the ∼100,000 nano-traps are continuously shifting forward along the fibre and the molecules trapped therein remain stationary in the lattice frame. This confers the OS-GPM a dynamics analogous to Wannier–Stark ladder structure^17^ used in condensed matter physics to explain for example quantum transport in crystals in the presence of a uniform electric field, or the effect of gravity in vertical cold atom lattices. Whilst a full quantum treatment is necessary to predict all the phenomena involved here, a simplified semi-classical approach is sufficient and justified by the fact that the trapped molecules of the OS-GPM are tightly kept attached to the lattice wells (strong tight-binding regime^17^) by the ultra-large potential depth (that is, 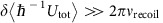). Within this strong tight-binding regime, the trapped molecules in each nano-trap of the lattice are isolated from those in the other wells, and their energy and motional dynamics are chiefly determined by the well depth and width. This is so even in the presence of the lattice acceleration, as the force 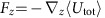 exerted on the lattice remains much smaller than the critical force 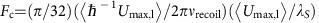 required for Landau–Zener tunnelling between adjacent nano-traps^17^. Consequently, and in consistence with the above observed SRS spectra, the lattice macroscopic acceleration does not induce major perturbation to the trapped molecule quantum states nor any Landau–Zener tunnelling between nano-sections^17^. The effect of such accelerating effect on these deeply trapped molecules is limited to a collective Doppler frequency shift between the forward and backward scattering, which is experimentally observable in the frequency spacing difference between FS and BS sidebands.

Figure 4b,c illustrates the interplay of the microscopic and macroscopic dimensions of our experiment (Supplementary Note 9). At the microscopic scale, the collision-free phase-space diagram in Fig. 4c reproduces the periodic and extremely narrow nano-traps (black sections in Fig. 4b) and their capture velocity of 1,800 m s^–1^ over a zoom-in segment of the fibre section FS2. Macroscopically, by tracing the molecules' trajectories shown in Fig. 4b, one can clearly see the total drift of the molecules towards the fibre end caused by the potential gradient, which reaches values up to 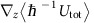
=−3,070 THz m^–1^, corresponding to ∼1 million times the gravitational acceleration on Earth. Here the molecules in the whole fibre undergo continuous flow forward, with velocity limited by viscosity. The overall laminar flow velocity^32^ was calculated to be ∼0.3 m s^–1^ using H_2_ viscosity value. This is qualitatively consistent with the value of 0.7 m s^–1^ deduced above from the Doppler shift between FS and BS for the molecules inside the moving lattice.

The consequence of this slow macroscopic motion is remarkably observable with the ‘naked eye'. Figure 4d shows selected frames from a video recording the side-scattered light from a 4 m-long HC-PCF section coiled in a spiral for the case of *P*_in_=25 W, and *p*_g_=20 bar, and situated at *z*∼2 m away from the fibre input (Supplementary Note 11 and Supplementary Movie 1). The video displays the motion of scattering nanoparticles (indicated by yellow circles) that are dragged along by the hydrogen flow with velocity of roughly 1 m s^–1^. The dependences of the scatters position on time for different pump powers indicate that the scatters move with a constant drift velocity, in agreement with theory (Supplementary Note 11).

## Discussion

The present configuration of trapping molecules and its rich results has the potential to provoke thoughts and ideas in further exploring theoretically and experimentally the underlined physics. For example, the strong localization of the molecules raises the question on the possibility of collective effects such as superradiance, or on the diffusion dynamics in this regime and its effect on the linewidth narrowing limit. Because the technique can be extended to a large number of Raman active molecules and atoms with a large Raman transition frequency, it will offer new approach and platform for quantum molecular physics and condensed matter.

The scheme can be used to engineer highly narrow-linewidth and powerful CW Raman lasers, optical micro-mirrors and micro-cavities made with gas-phase materials. As shown, one can generate high power and scalable CW Raman generator with ultra-narrowed gain by simply adjusting the fibre length and a Raman active gas pressure. Also, because the present molecular optical lattice is associated with a strong index modulation, the lattice acts as a strong mirror, and in turn one can engineer two these novel optical lattices at different sections of a gas-filled HC-PCF to form a cavity inside the fibre solely made out of a gas material.

Finally, the phenomena for investigations in the immediate future include finding out whether this scheme could lead to molecular cooling via the observed resolved motional sidebands. Such investigation could be undertaken by adding to the pump laser two lasers whose frequency is red-detuned from the pump by the transverse and longitudinal motional frequency, respectively. This would enable engineering molecular non-classical state, and entangled photons to mention a few. Finally, the large acceleration in this laser-gas interaction could be explored as a new tool for micro- and nanoparticle accelerators.

### Data availability

The data that support the findings of this study are available from the corresponding author on request.

## Additional information

**How to cite this article:** Alharbi, M. *et al*. Raman gas self-organizing into deep nano-trap lattice. *Nat. Commun.* 7:12779 doi: 10.1038/ncomms12779 (2016).

## Supplementary Material

Supplementary Information Supplementary Figures 1-17, Supplementary Notes 1-11 and Supplementary References

Supplementary Movie 1 Moving molecules
