# Supplementary material for: Raman gas self-organizing into deep nano-trap lattice
Source: Nat Commun. 2016 Sep 28;7:12779. doi: 10.1038/ncomms12779 (PMC5052700; doi:10.1038/ncomms12779)
Supplement: Supplementary Information — Supplementary Figures 1-17, Supplementary Notes 1-11 and Supplementary References [file ncomms12779-s1.pdf]

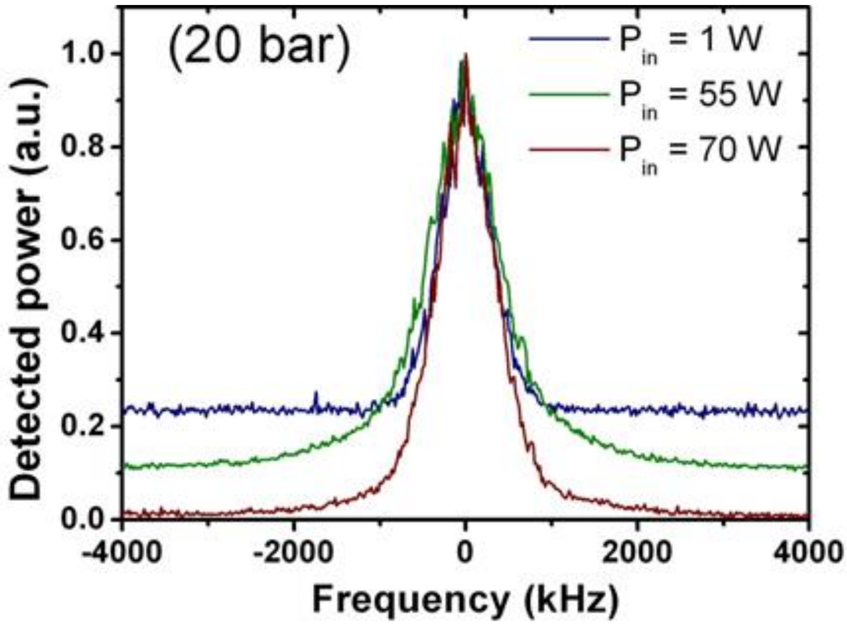

Supplementary Figure 1. Pump linewidth for different input power at a pressure of 20 bar and fibre length of 20 m

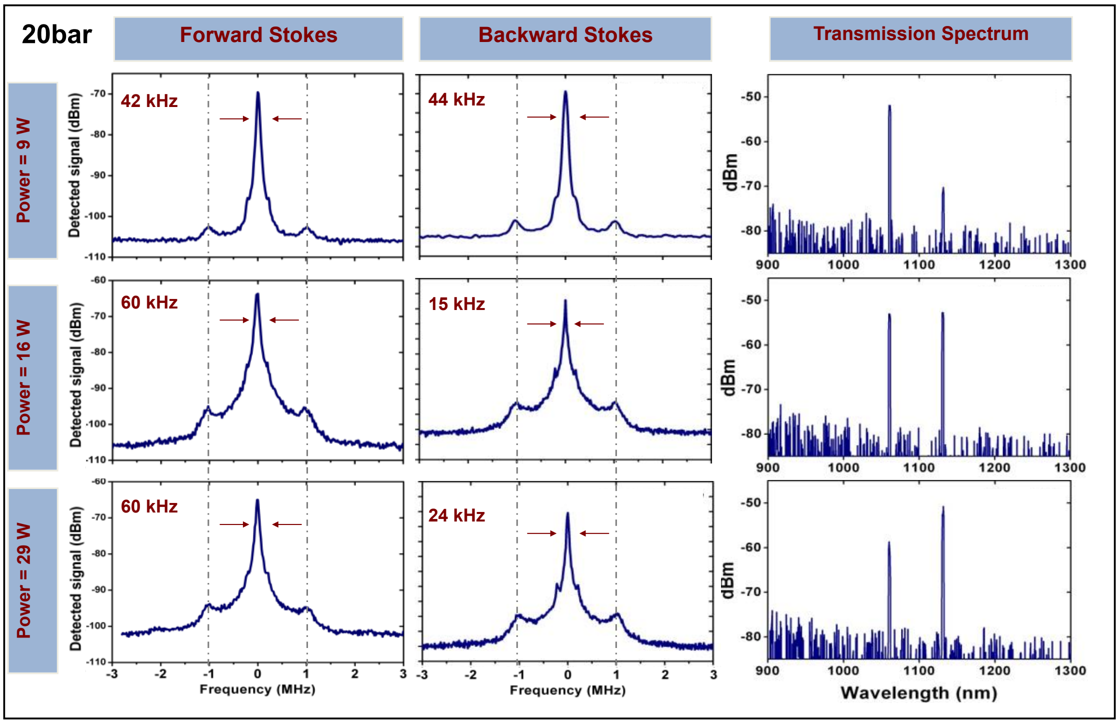

Supplementary Figure 2. Linewidth traces of FS (first column) and BS (second column) and the optical spectral of the transmitted laser beam for different input power at a pressure of 20 bar and fibre length of 20 m. The measured linewidth is indicated in red.

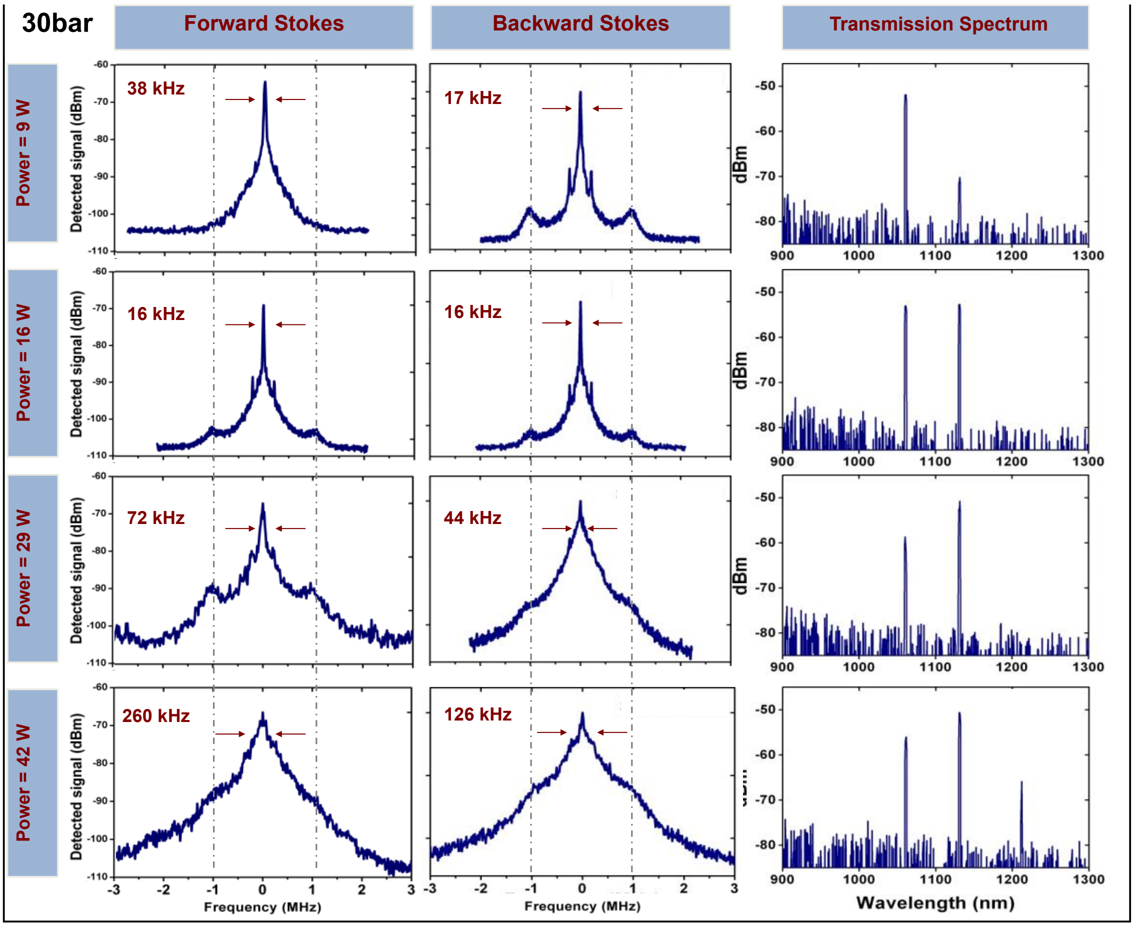

Supplementary Figure 3. Same as in supplementary figure 2 but for a gas pressure of 30 bar.

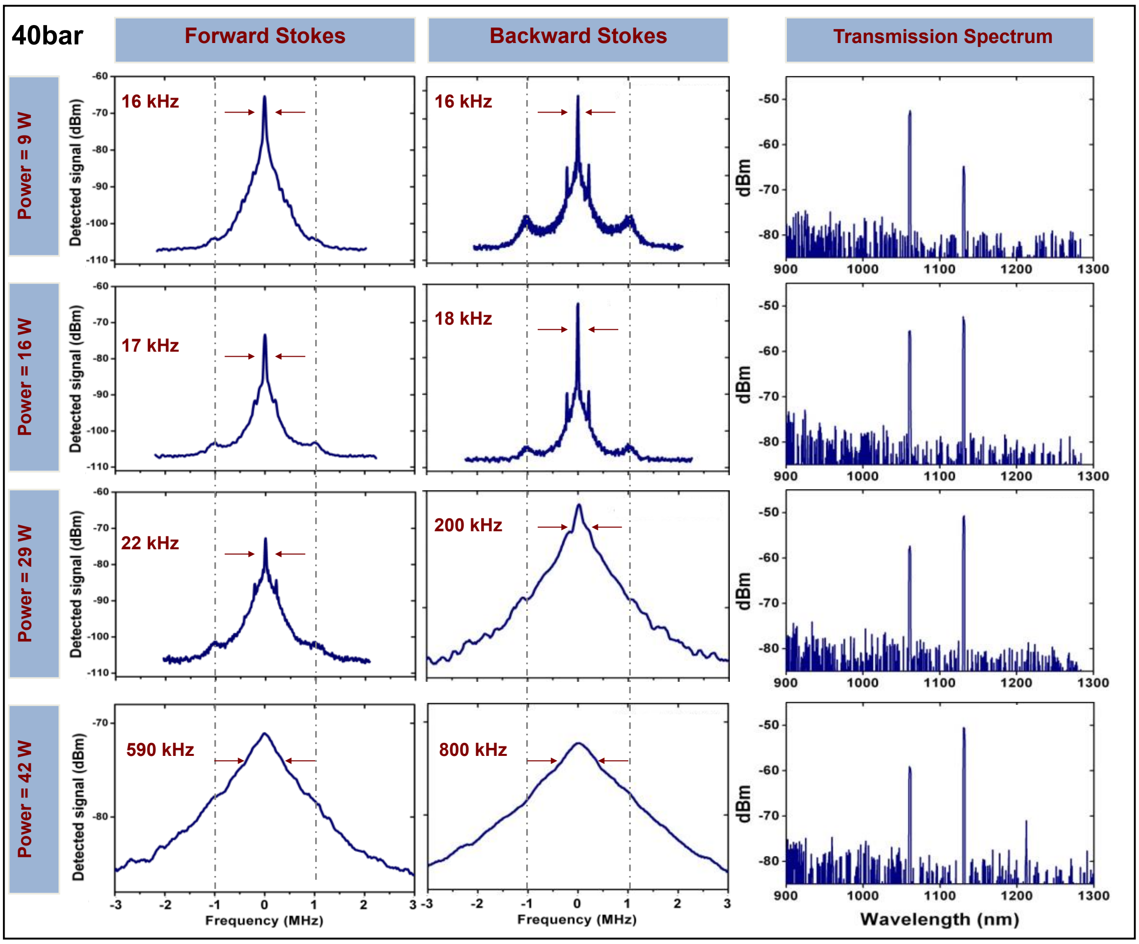

Supplementary Figure 4. Same as in supplementary figure 2 but for a gas pressure of 40 bar.

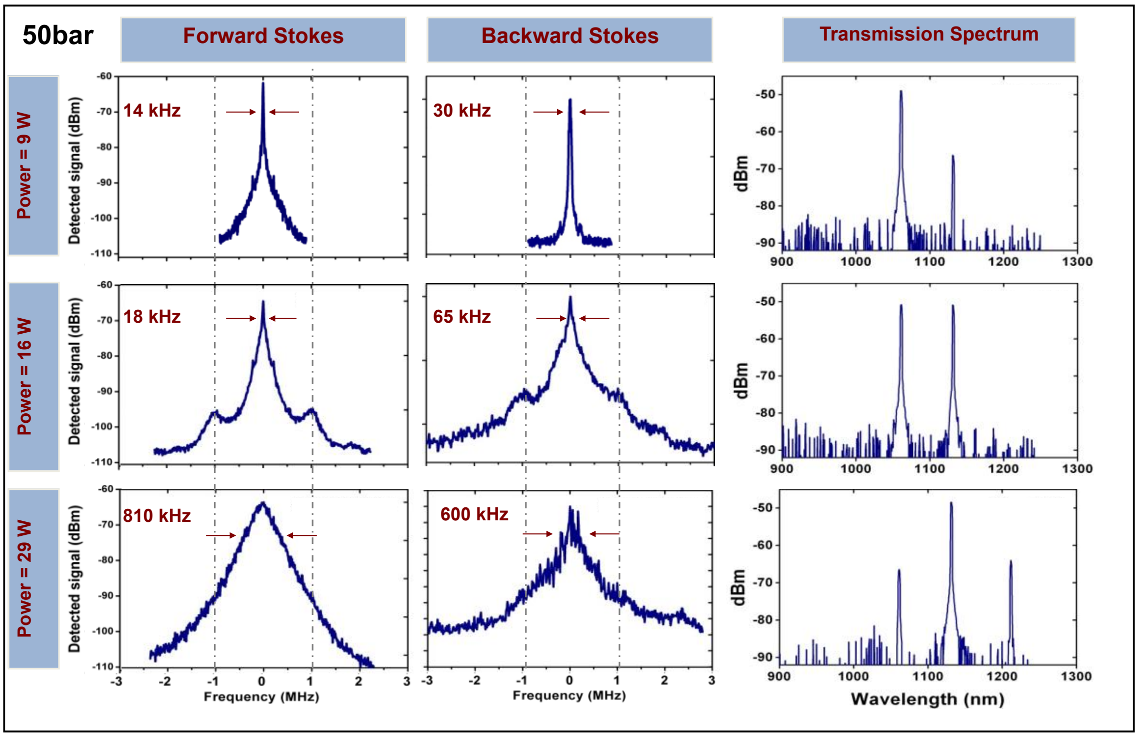

Supplementary Figure 5. Same as in supplementary figure 2 but for a gas pressure of 50 bar.

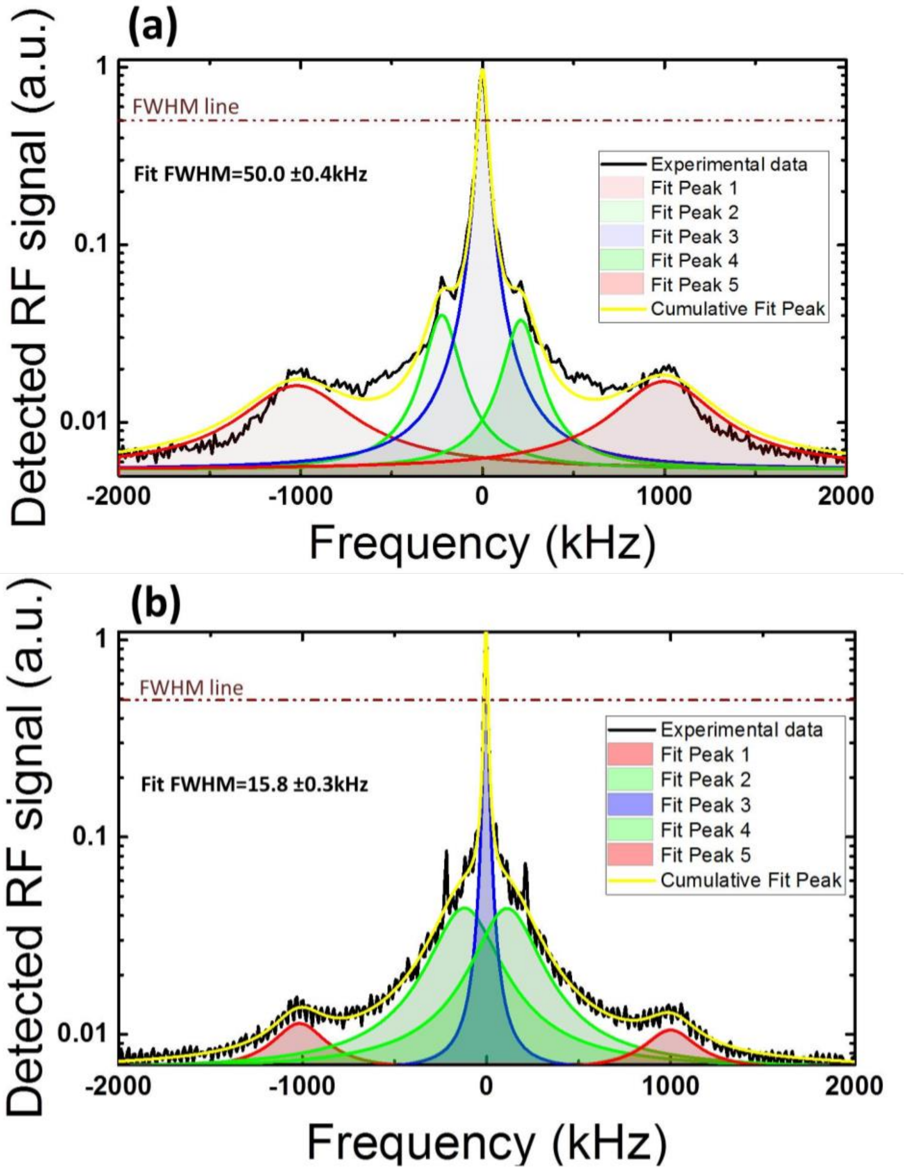

Supplementary Figure 6. Forward Stokes RF spectrum and its nonlinear fit for a pump power and pressure of 9 W and 20 bar (a) and 16 W and 30 bar (b) respectively.

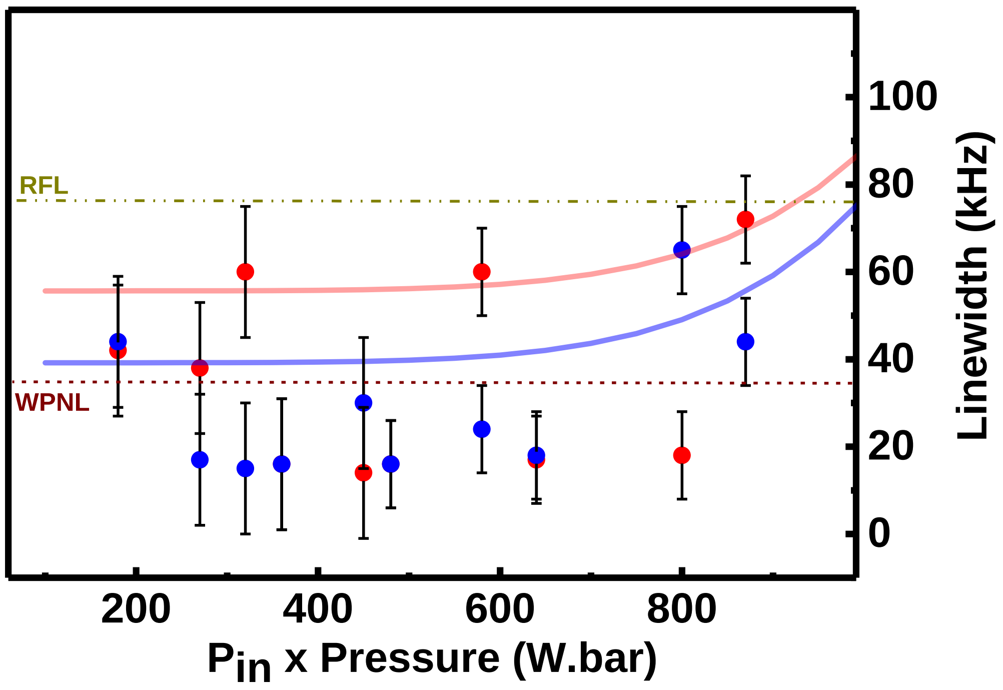

Supplementary Figure 7. Evolution with the product of pump input power and pressure of the linewidth of forward Stokes (blue points) and backward Stokes (red points). The solid lines are a fit for eye-guidance for the linewidth evolution of the forward Stokes (blue line) and backward Stokes (red line). Here the pump power and pressure range was limited to the case where the 2nd Stokes is not generated.

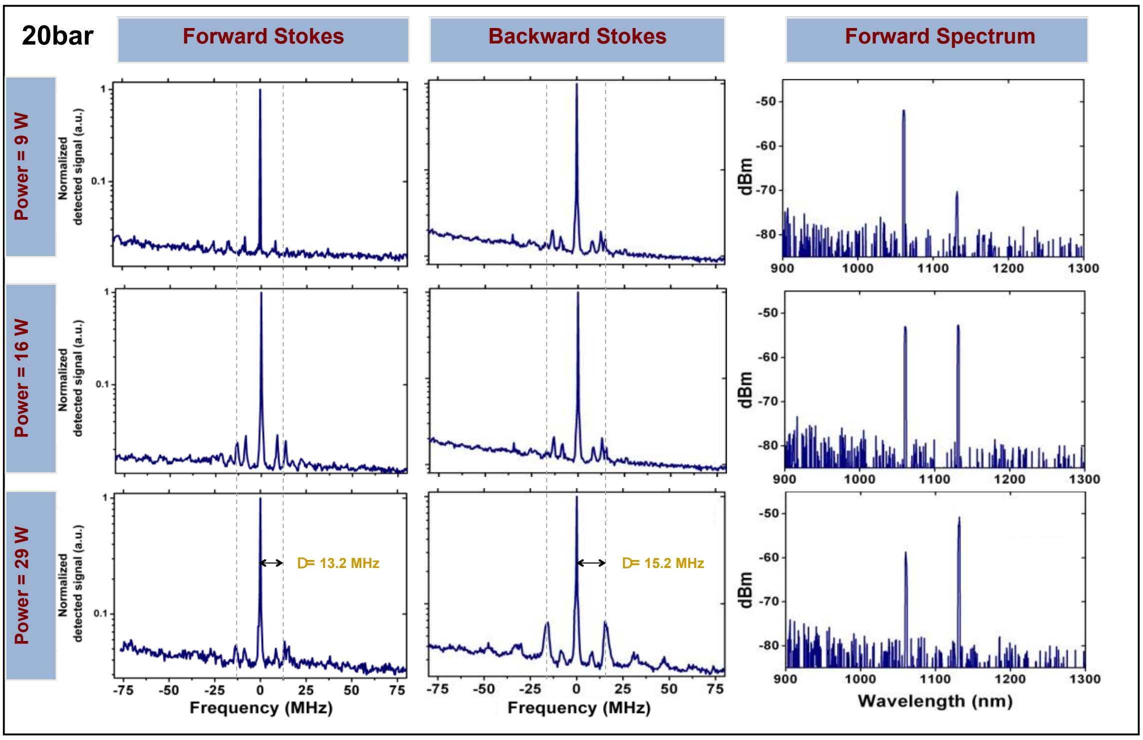

Supplementary Figure 8. 150 MHz span RF-spectra of Forward Stokes (first column) and Backward Stokes (second column) and the optical spectral of the transmitted laser beam (third column) for different input powers at a pressure of 20 bar and fibre length of 20 m. The measured two photon Rabi frequency is indicated for one representative case.

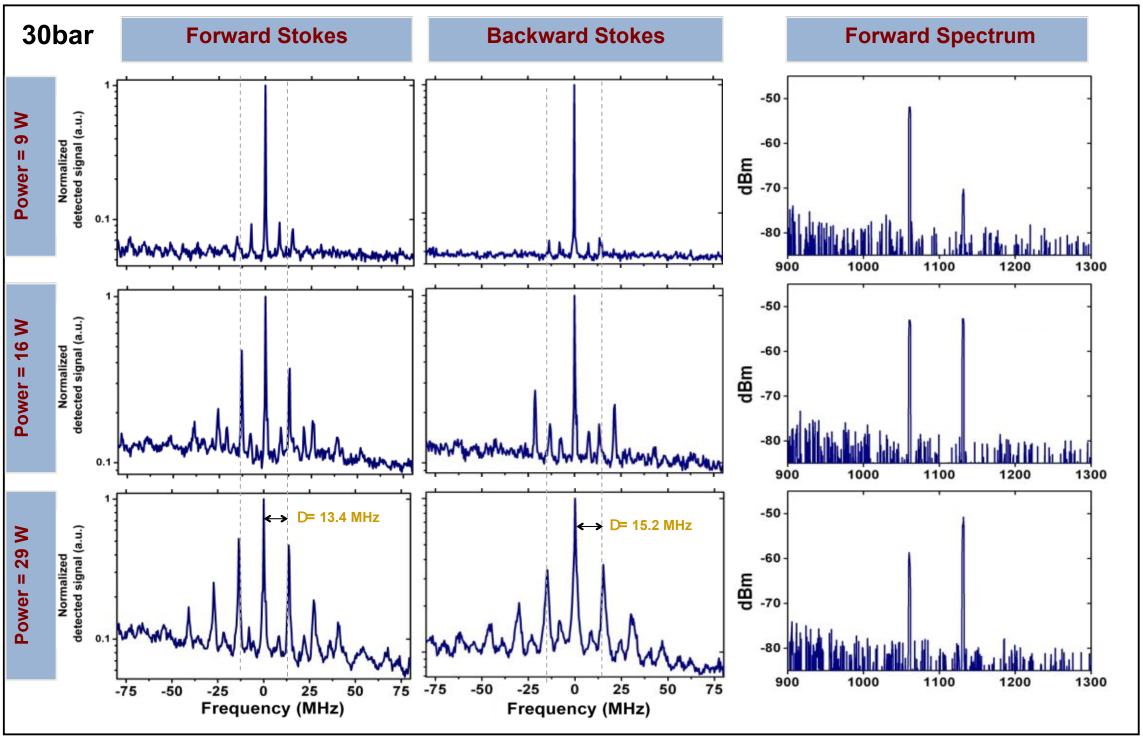

Supplementary Figure 9. Same as in supplementary figure 8 for gas pressure of 30 bar.

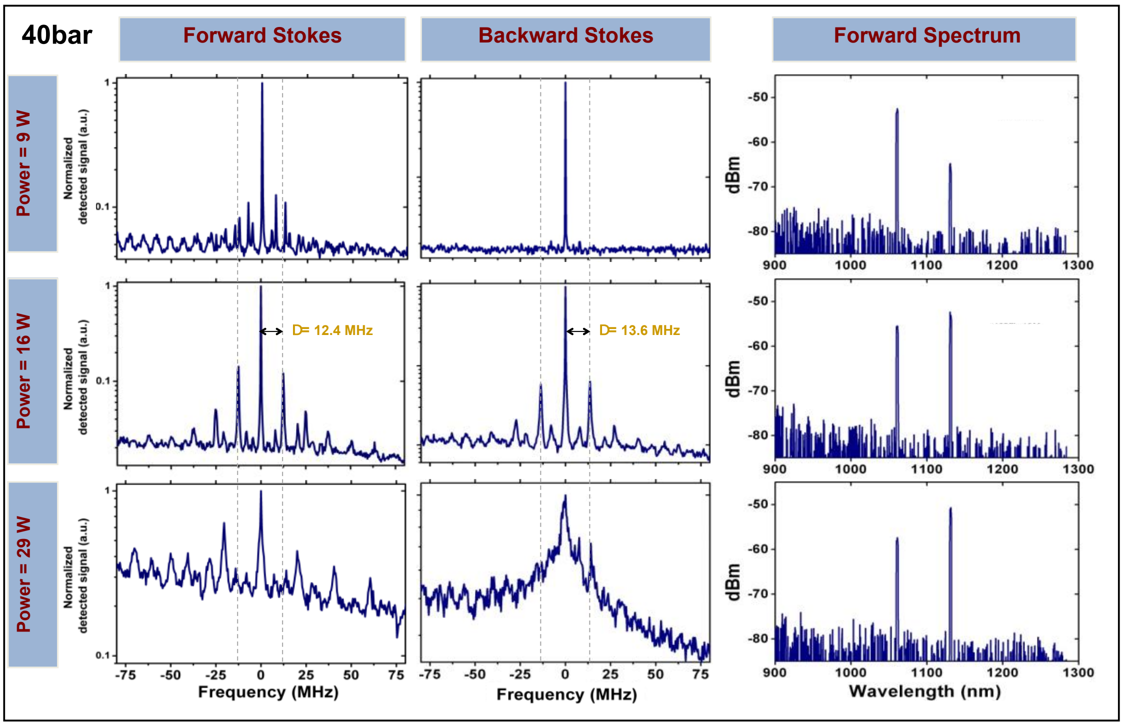

Supplementary Figure 10. Same as in supplementary figure 8 for gas pressure of 40 bar.

.

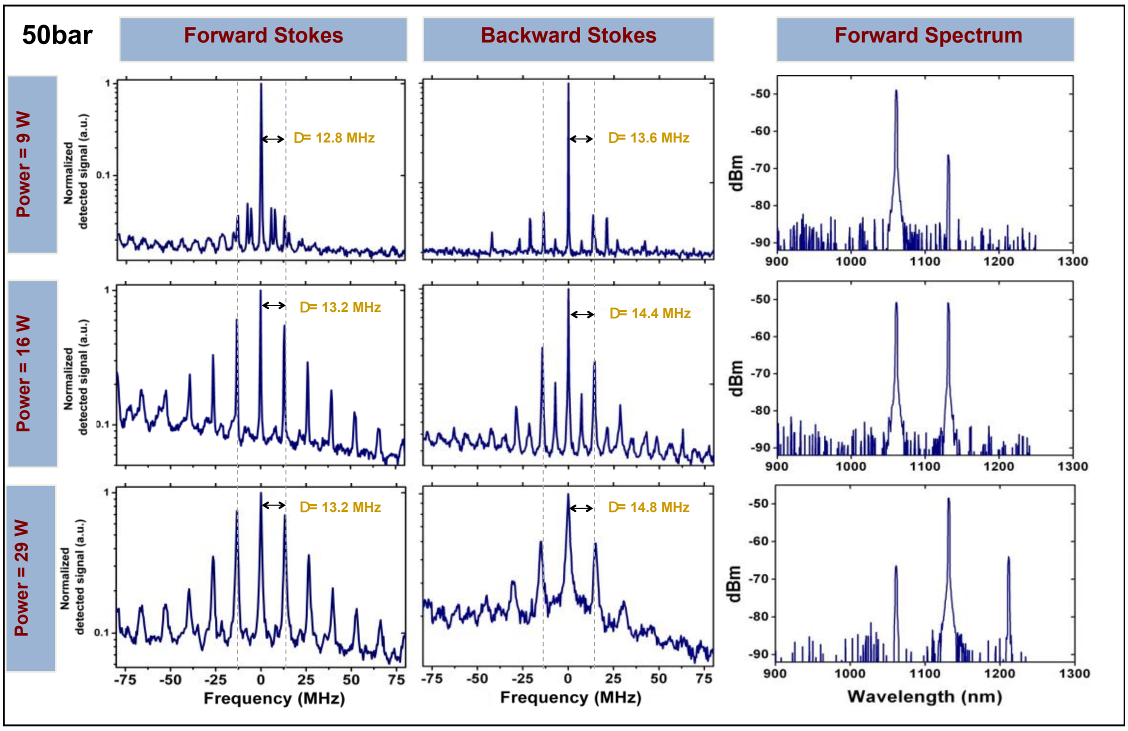

Supplementary Figure 11. same as in supplementary figure 8 for gas pressure of 50 bar.

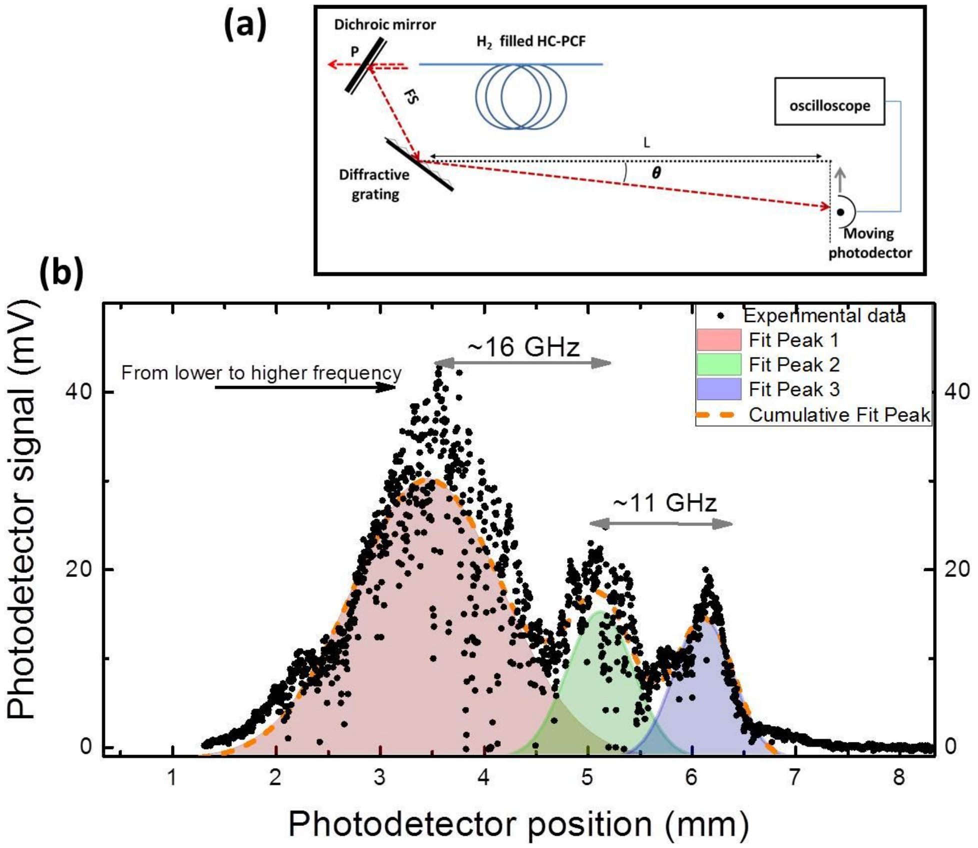

Supplementary Figure 12. Experimental set-up to measure the longitudinal-motion sidebands. The forward Stokes beam is diffracted using a diffractive grating with a groove spacing of 833 nm, and is recorded using a photodetector placed at a distance L=11 m from the diffractive grating. The input pump power was ~20 W, and the gas pressure: 15 bar.

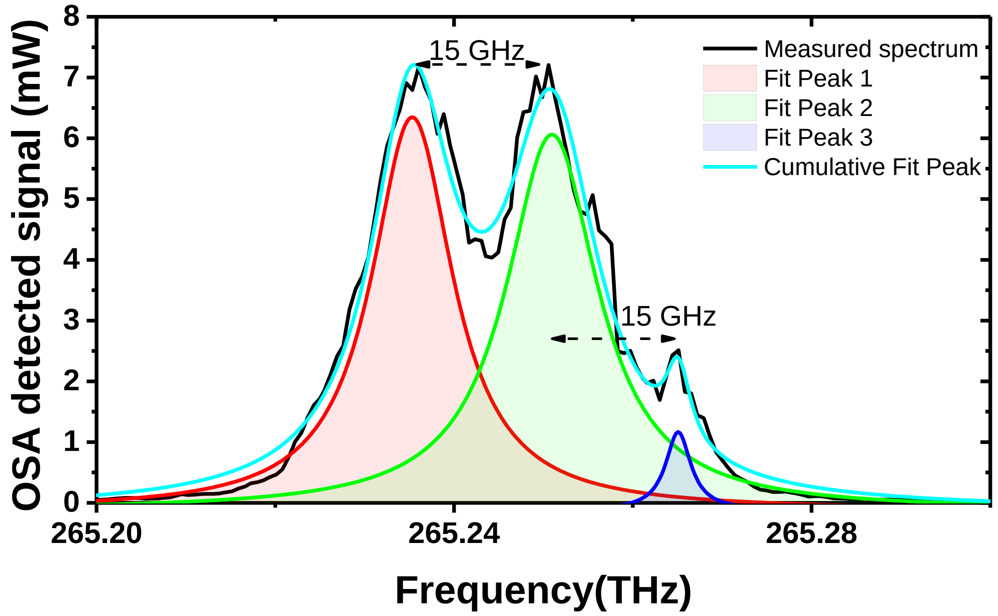

Supplementary Figure 13. Measured spectrum of FS at input pump power of ~20 W and pressure of 15 bar. The shaded curves are the multiple Gaussian peaks fit.

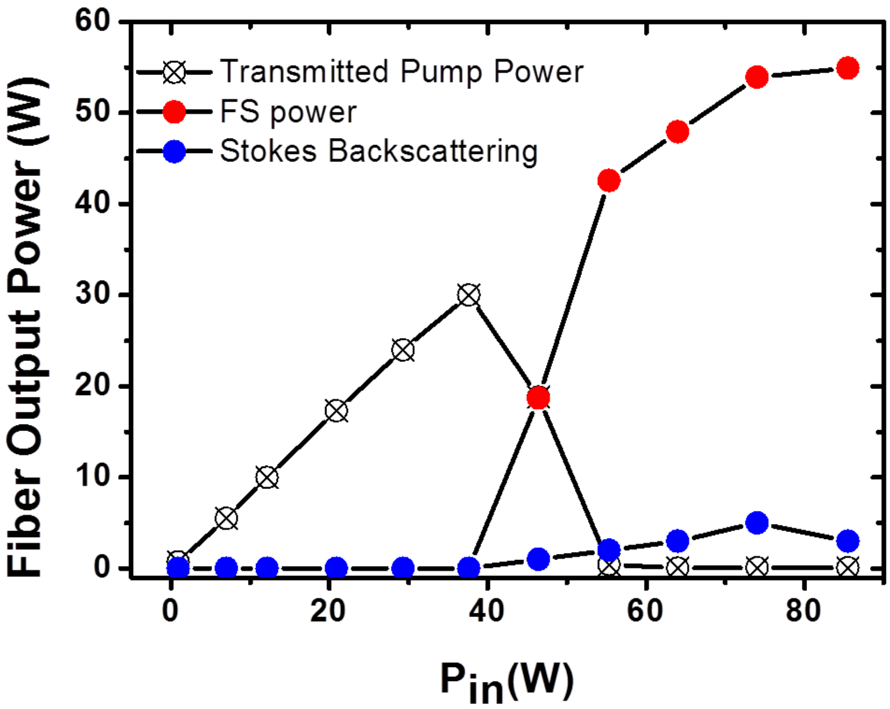

Supplementary Figure 14. Pump, FS and BS power evolution with input power for a fibre length of 7 m and a gas pressure of 20 bar.

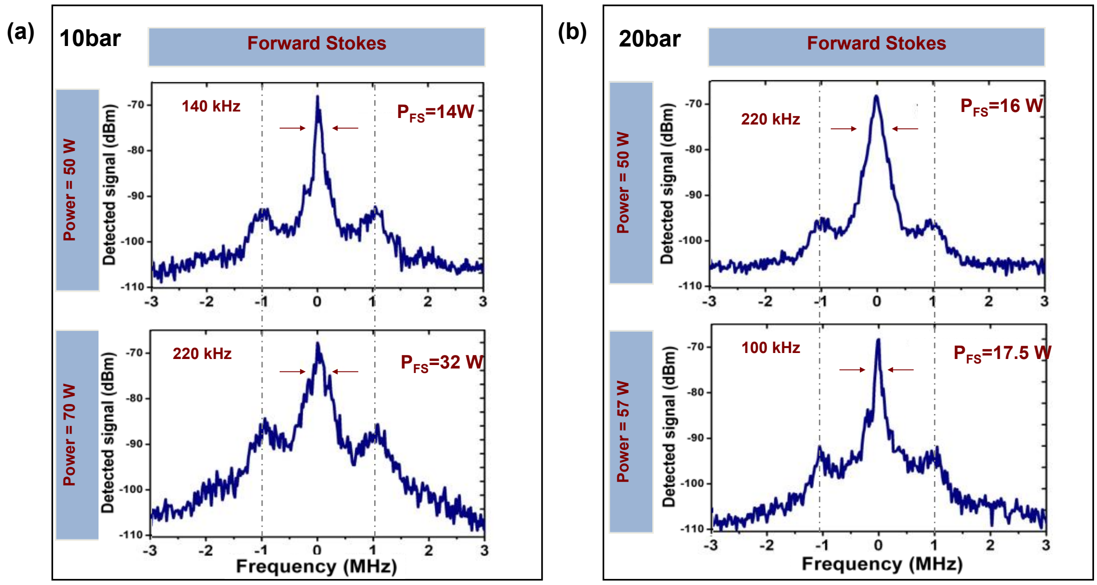

Supplementary Figure 15. (A) Linewidth for input power above 50 W for FS and BS using 7 m of PBG HC-PCF at gas pressure of (a) 10 bar and (b) 20 bar.

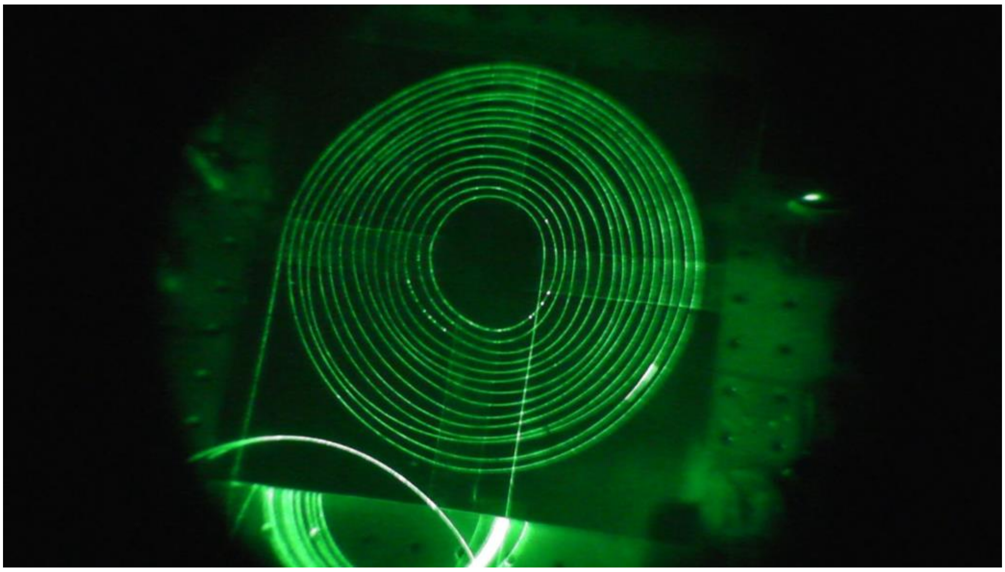

Supplementary Figure 16. A snapshot of a recorded video for moving molecules at input power of ~ 29W (video available online).

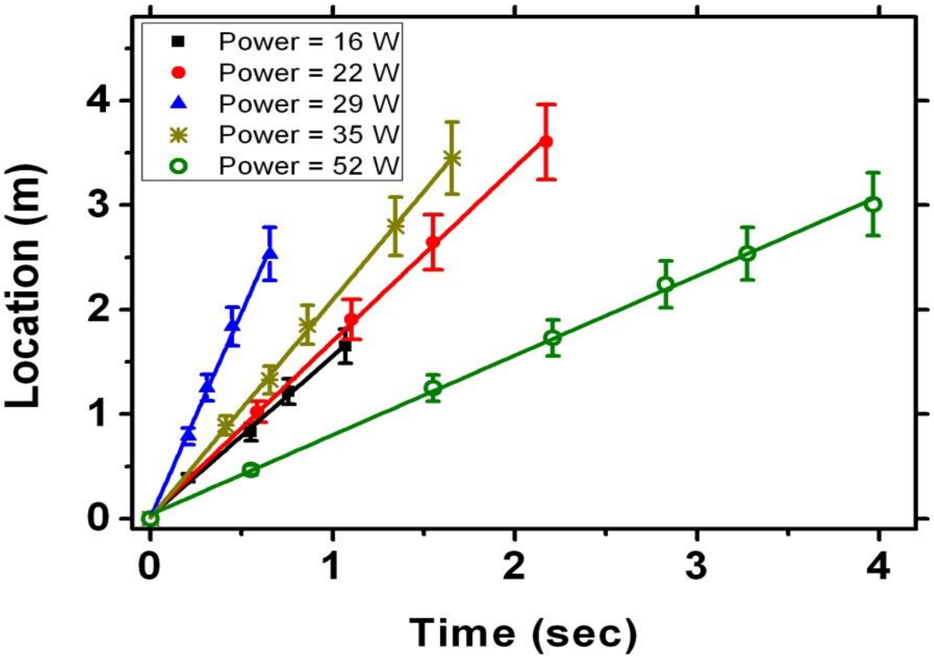

Supplementary Figure 17. Scatter's location evolution with time for different input pump

Supplementary note 1: Experimental set-up for optical and RF spectral measurements.

The 20 m long fibre used here is a home-made photonic bandgap (PBG) guiding HC-PCF and fabricated using the stack and draw technique. The fibre is filled with molecular hydrogen at a controllable pressure, by placing the two fibre-ends in gas cells. The gas pressure is kept uniform along the whole length of fibre by monitoring it with pressure gages placed at both cells. The gas cells are equipped with AR coated windows at both sides to avoid laser back reflection. The fibre has a core radius of r = 3.2 µm (see top of Fig. 1B), and guides from 1000 nm to 1200 nm (Fig. 1C, red curve in the main manuscript), with a loss of 70 dB/km. The choice of PBG HC-PCF is motivated by its narrow transmission bandwidth, which favours the SRS conversion to the 1st order Stokes of H_2 rotational transition. This is achieved by eliminating the vibrational SRS, as its first-order Stokes and anti-Stokes frequencies lay outside the fibre transmission window. Furthermore, the frequencies of the 1st order anti-Stokes and second-order Stokes of the rotational SRS are close to the transmission window edges, and hence exhibit much higher transmission loss than the pump and 1st Stokes rotational frequencies.

The hydrogen-filled fibre is pumped with a randomly polarized 1061 nm wavelength Yb-fibre CW laser that could emit up to a maximum of 100 W of optical power, with a linewidth of only ~400 kHz. The optical spectrum from both fibre-ends is monitored using an optical spectrum analyser to record the FS and BS spectra.

Furthermore, the experimental set-up also comprises a portion with a delayed self-heterodyne interferometer to measure the linewidth of both forward and backward propagating beam spectral components. The self-heterodyne system consists of a delay arm made of a 6 km long optical single-mode fibre (SMF) at wavelength longer that 1 µm, and a short modulation arm comprising an acousto-optic modulator (AOM) operating at ~211 MHz. The beat signal between the delayed optical beam and the AOM-frequency down-shifted signal is detected using a fast photo-detector (~1 GHz bandwidth) and recorded using an RF spectrum analyser (Rohde&Schwarz FSEA 30). The RF spectrum analyser resolution bandwidth is set at 10 KHz when the span bandwidth is ~150 MHz, and 1 kHz for a narrower span of less than 6 MHz. The RF spectral traces of the pump, FS and BS are

then recorded for different pump laser powers and gas pressures over the two above mentioned bandwidth span.

Supplementary note 2: Pump linewidth spectrum

The linewidth of the transmitted residual pump has been monitored for different input pump powers and gas pressures. Supplementary figure 1 shows that the pump linewidth remains unchanged with input power increase.

Supplementary note 3: The theoretical model

We consider the propagation of continuous-wave pump and first order Stokes radiation in the fundamental mode of a photonic bandgap guiding photonic crystal fibre, neglecting the excitation of higher-order Stoke and anti-Stokes lines, as well as the energy transfer to higher-order transverse modes of the fibre.

The propagation characteristics of Stokes and pump, such as the wavenumbers β_S, β_p as well as losses α_s, α_p, were calculated using the JCMwave finite-element Maxwell solver with high precision, using the tabulated data of the fused silica refractive index and the transverse cross-section of the fibre determined by the microscopy.

To derive the propagation equations, we first consider the steady-state values of the density matrix ρ, which are given through the coherence ρ_{12} and the population difference $D = \rho_{22} - \rho_{11}$, where *1* and *2* denote the ground and excited rotational states of the H_2 molecules. At a fixed position, the electric fields of Stokes and pump components are given by $E_S(t) = (1/2)E_S e^{-i\omega_S t} + c.c.$ and $E_p(t) = (1/2)E_p e^{-i\omega_p t} + c.c.$ The steady-state values in this case are:

$$D = -\frac{\Gamma_{12}\gamma_{12}{}^2}{\Gamma_{12}\gamma_{12}{}^2 + 4|\Omega_{12}|^2\gamma_{12}} \tag{1}$$

$$\rho_{12} = \frac{i\Omega_{12}D}{\gamma_{12} - i(\Omega_{11} - \Omega_{22})}, \tag{2}$$

where the Rabi frequencies Ω_{11}, Ω_{22} and Ω_{12} are defined by

$$\Omega_{11} = 0.5(a_p|E_P|^2 + a_s|E_S|^2), \tag{3}$$

$$\Omega_{22} = 0.5(b_p|E_P|^2 + b_s|E_S|^2), \tag{4}$$

$$\Omega_{12} = 0.5 d_s E_P^* E_S. \quad (5)$$

Here a_s, b_s, a_p, b_p, d_s being constants related to dipole moments. The quantities Γ_{12} and γ_{12} are the population decay rate and the Raman gain linewidth, correspondingly.

The Stokes component in the considered case consists of the forward-propagating part $E_S^{(f)}$ and backward-propagating part $E_S^{(b)}$, with $E_S = E_S^{(f)} + E_S^{(b)}$, which satisfy two distinct propagation equations. The values ρ_{12} and D allow calculating the polarizations P_{SF}, P_{SB} and P_P as

$$P_S{}^{(f)} = 2N\hbar(a_S\rho_{11} + b_S\rho_{22})E_S{}^{(f)} + i\frac{N\hbar^2 d_S^2 D}{\gamma_{12} - i(\Omega_{11} - \Omega_{22})}|E_P|^2 E_S{}^{(f)}, \quad (6)$$

$$P_S{}^{(b)} = 2N\hbar(a_S\rho_{11} + b_S\rho_{22})E_S{}^{(b)} + i\frac{N\hbar^2 d_S^2 D}{\gamma_{12} - i(\Omega_{11} - \Omega_{22})|}E_P|^2 E_S{}^{(b)}, \quad (7)$$

$$P_P = 2N\hbar(a_P\rho_{11} + b_P\rho_{22})E_P + i\frac{N\hbar^2 d_S^2 D}{\gamma_{12} - i(\Omega_{11} - \Omega_{22})|}E_S|^2 E_P, \quad (8)$$

where N is the concentration of the molecules.

The propagation equations then are written as

$$\partial_z E_S{}^{(f)} = -\frac{\kappa\omega_S}{2c\varepsilon_0}P_S{}^{(f)} - \alpha_S E_S{}^{(f)}, \quad (9)$$

$$\partial_z E_S{}^{(b)} = -\frac{\kappa\omega_S}{2c\varepsilon_0}P_S{}^{(b)} + \alpha_S E_S{}^{(b)}, \quad (10)$$

$$\partial_z E_P = -\frac{\kappa\omega_S}{2c\varepsilon_0}P_P - \alpha_P E_P, \quad (11)$$

where $\kappa = \kappa(E_P, E_S^{(f)}, E_S^{(b)})$ is the parameter which accounts for the microscopic spatial distribution of the gain. In the propagation equations, we have ignored the change of the refractive index which arises from microscopic density modulation, as detailed below. The analytical expression for κ is cumbersome and is not given here.

The origin of the backward Stokes component is the reflection from the input and output fibre interfaces back into the fibre due to mismatch of the effective refractive index of the fibre mode and of the free space, imperfections at the fibre ends, grating formed by the modulation of particle density, and fibre roughness, with a reflection coefficient estimated by a total value of 1% in energy. Therefore, the boundary conditions for the Stokes field are

$$\left|E_S{}^{(f)}(0)\right| = \sqrt{r}\left|E_S{}^{(b)}(0)\right|, \tag{12}$$

$$\left|E_S{}^{(b)}(L)\right| = \sqrt{r}\left|E_S{}^{(f)}(L)\right|, \tag{13}$$

where r is the energy reflection coefficient and L is the fibre length. We note that the model doesn't take into account the reflection from the molecular lattice index modulation. Although neglecting the reflection off the Stokes optical lattice will have an effect on the exact magnitude of FS and BS, it doesn't significantly impact the results reported here.

Above equations were solved self-consistently with the propagation equations, using the numerical shooting method to determine $E_S{}^{(f)}(0)$. No reflection of the pump field was considered, since the values of the pump field at the fibre output were typically quite low. The following input parameters were assumed: ω_S = 1.6838 fs^{-1}, ω_P = 1.7933 fs^{-1}, a_p = 3.854x10^{-7} m^2/s/V^2, a_s = 3.849x10^{-7} m^2/s/V^2, b_p = 3.85831x 10^{-7} m^2/s/V^2, b_s = 3.8536x10^{-7} m^2/s/V^2, d_s = 3.8538x10^{-7} m^2/s/V^2, γ_{12} = 2π (1.14x10^9) Hz, Γ_{12} = 2π(2 x10^5)Hz, N = 4.86x10^{26} 1/m^3 at the pressure of 20 bar, L = 20 m, and waveguide core radius is 3.2 μm.

In our model, we consider the frequency difference between the pump and scattered Stokes to be equal to the Raman transition frequency, and the initial population is in the ground state (i.e. $D = -\rho_{11} = -1$).

Supplementary Note 4: FS and BS linewidth spectrum over narrow spectral span

Fig. 3B of the main manuscript summarizes the results of FS and BS spectral emission linewidth and structure over ± 2 MHz span along with the transmitted optical spectrum. The results are extracted from the full data set shown in supplementary figures 2-5. Each figure shows, for a given fixed gas pressure, the evolution with the pump input power of both the optical spectrum of the transmitted beam, and the RF spectrum of the forward and backward Stokes lines over a 6MHz-span. Here, the input power ranges from 9 W (i.e. coupled power of 5.4 W) to 42 W. Given the necessity of resetting the laser fibre-coupling for each power level due to the change of its beam size and divergence, the number of input power runs was limited to 3 or 4 power-values of 9 W, 16W, 29W and 42 W. The figure series show the same content but at a different pressure. Four pressure values of 20 bar, 30 bar, 40 bar and 50 bar were investigated.

For a pressure and pump power range corresponding to the absence of 2nd Stokes, all the RF spectral data exhibits 4 lateral peaks and 1 strong central peak, with a ratio relative to the background floor larger than 35 dB. The traces have been recorded several times (typically in the range of 10-15 times) for each value of pressure and pump input power. This data set was used to extract the linewidth magnitude shown in Fig. 3(B) of the main manuscript text. This was carried out by performing a nonlinear fit of the lineshape over 4 MHz span, and extracting the linewidth parameter of the central line.

Supplementary figure 6 shows typical fitted spectra for two different sets of pressure and input pump power. Supplementary figure 6 (a) corresponds to a pump power of 9 W and a pressure of 20 bar, while supplementary figure 6 (b) corresponds to a pump power and a pressure of 16 W and 30 bar respectively. The nonlinear fit consists of 5 Lorentzian peaks and the results show a χ^2 goodness fit value larger than 0.996 for all the fitted spectra. In addition, we repeated the fit using the pseudo-Voigt function instead of Lorentzian and found linewidth within 5% discrepancy compared to Lorentzian fit. In the fit, we chose to not fully resolve the centre of the peaks located in the range of ±215-225 kHz (depending on the pump power and pressure) as they are extremely narrow, which is consistent with Lamb-Dicke narrowing of motional sidebands. The goodness of the fit confirms the validity of our measurements. It is noteworthy that evaluation of the experimental points which are spaced by more than 34.5 kHz from the line center show very good signal-to-noise ratio and fit very well. This permits us to draw definite conclusion on the linewidth, based also on the experimental data far from the line center. For each pair value of pump power and pressure, we calculate the mean value and standard deviation from the extracted linewidth, which are plotted in supplementary figure 7. The uncertainty in the linewidth is chiefly due to the pump power coupling fluctuation due to the heating effect of the fibre holding cell. Furthermore, this supplementary figure shows the recoil frequency limit (labelled RFL in the supplementary figure), and all the measured linewidths are below this limit, for the shown range of pressure and pump power.

The horizontal line at ~34.5 kHz, in supplementary figure 7, corresponds to the white phase noise limit (labelled WPNL in the supplementary figure) set by the fibre length of 6 km on the minimum measurable linewidth where the effect of phase correlation between the beat signals is strongly suppressed. This limit is given by $\Delta\nu_{\mathrm{WPNL}} = c/(n\, L_{\mathrm{fib}})$ and is inversely proportional with the fibre length in the delay arm of the unbalanced interferometer. Here c is the speed of light, n is the fibre

core material index and L_{fib} is the fibre length. A large fraction of the measured linewidths with was found to be lower than $\Delta\nu_{\mathrm{WPNL}}$, which raises questions on the accuracy or validity of the measured linewidth for the values below 34.5 kHz. To address this question, we first recall that this rule is only valid for a white frequency noise where the phase noise is assumed to be a zero-mean stationary random Gaussian process, which is not necessary the case of our experiment, where the Stokes radiations are emitted in a Lamb-Dicke regime. For a non-white phase noise structure, the above limit condition doesn't necessarily hold. Furthermore, reported works [1,2] show that linewidth as low as 6 kHz could be measured with a fibre length of only 2 km long by extracting the spectral line-shape from the phase spectral noise (PSD). We compared this PSD technique of line-shape extraction to our nonlinear fit by reconstructing the line-shape of the beat signal from its temporal trace (recorded by an oscilloscope with 2 ms integration time) and comparing it to the nonlinear fitted RF spectrum. We found that the two techniques give similar results within less than 10% of relative discrepancy. In order to further ensure the validity of our linewidth measurements, for values less than 34.5 kHz, we have estimated our fittings for spectral data points with frequency offset from the centre in the range of 50 to 200 kHz to avoid the interferometer effect on the linewidth. We found that all the experimental data at the RF frequency range exhibits a strong signal to noise ratio and fit extremely well with the multiple peak Lorentzian function. A final test on the validity of the linewidth measured was done by proceeding with linewidth measurements with different delays, and we found that the linewidth values are independent with delay variation within 20%.

Supplementary Note 5: FS and BS linewidth spectrum over a broader spectral span

Here, we re-examine the linewidth traces and their evolution with input power and gas pressure, but over a larger RF frequency span of 150 MHz (resolution of 400 kHz). The recorded spectral traces are displayed in a similar fashion to those of the linewidth fine structure. Four supplementary figures (Supplementary figures 8-11) contain the evolution of the spectral traces with input power for a given fixed pressure. The pressure values are the same as in Supplementary note 4. For the lower-range pressure (20 bar), the spectral traces of both FS and BS are dominated with a single narrow peak, which are accompanied by mainly two families of sidebands. The first sidebands are located in the range of Δ~12-15 MHz. Their harmonic frequencies are identified as the two photon Rabi

sidebands (see main manuscript). These sidebands vary little with input power and gas pressure due to the fact that the Raman active molecules are limited to those located in nano-traps. The definition of the two photon Rabi frequency is given in Supplementary note 8.

Furthermore, we observe that FS and BS exhibit a difference in the frequency of their sidebands in the range of sideband frequency of 1.2-2 MHz due to the overall motion of the molecular lattice. The second family of sidebands are located in the range of ~7-8 MHz, which is roughly half that of the Rabi sideband frequency. Moreover, higher order Rabi sidebands are also observed. We attribute this effect to four wave mixing (FWM) between Stokes central peak and the two sidebands and with a frequency given by $\omega_{\mathrm{2nd}} = \omega_{\mathrm{S}} + (\omega_{\mathrm{S}} \pm \Omega_{\mathrm{Rs}}) - (\omega_S \mp \Omega_{\mathrm{Rs}})$=$(\omega_{\mathrm{S}} \pm 2\Omega_{\mathrm{Rs}})$. The 2nd order Rabi sideband signal will be determined by the nonlinear susceptibility at its frequency $\chi(\omega_{2nd})$ via $E_{\mathrm{2nd}} = \chi(\omega_{\mathrm{2nd}})E_{\mathrm{S}}(\omega_{\mathrm{S}})E_{\mathrm{Rs}}(\omega_{\mathrm{S}} \pm \Omega_{\mathrm{Rs}})E_{\mathrm{Rs}}^{*}(\omega_{\mathrm{S}} \mp \Omega_{\mathrm{Rs}})$.

Supplementary Note 6: Longitudinal-motion sidebands

The model predicts the existence of longitudinal-motion sidebands corresponding to the trapped molecules oscillation with the quasi-harmonic oscillator along the z-direction. The expression of the fundamental frequency of these oscillations is $\nu_{\mathrm{long}} = 2\left(\Omega_{12}^{(0)}/\Omega_{12}^{(sat)}\right)\sqrt{\nu_{\mathrm{recoil}}\langle\hbar^{-1}U_{\mathrm{max,l}}\rangle/\pi}$. For the ranges of pump power and gas pressure we explored, this expression predicts values from ~3 GHz to ~17 GHz. Such frequency values are outside our detection scheme. In order to prove experimentally their existence, we have proceeded with two measurement set-ups. First, we sent the beam of the forward Stokes (FS) to a diffractive grating with a groove spacing of 833 nm, and reconstruct its intensity profile along a given of a plane, which is 11 m away from the grating. Supplementary figure 12 (a) shows this set-up schematically. The photodetector is placed on a motorised linear stage to scan the beam at a constant speed. Supplementary figure 12 (b) shows the recorded trace of the photodetector detected signal as a function of its position for a pump power of 20 W and a pressure of 15 bar. The trace clearly shows three peaks spaced by around ~1.5 mm and ~1 mm corresponding to a spectral position of 16 GHz and 11 GHz respectively. Given the measurement uncertainty of our set-up, this is a very good agreement with the theoretical predictions.

Furthermore, the trace shows asymmetry in the peaks heights, which shows the quantum nature of the motion. Further work and more detailed measurements with appropriate instruments are needed to analyse the spectral structure of these sidebands. The second set-up to corroborate the longitudinal sideband consists of sending the FS to an optical spectrum analyser (OSA) set at its highest sensitivity and resolution (10 GHz). We note, however, that when the OSA is set on high sensitivity, the real resolution of our OSA is probably below 10 GHz based on our previous experience with this tool. Supplementary figure 13 shows the typical spectrum with its multiple peaks fit from several recorded spectra. Similarly with the first set-up results, the spectrum shows three distinct peaks. Here, the frequency spacing is equal between the peaks and found to ~15 GHz, in qualitative agreement with the above results despite the insufficient resolution of the OSA.

Supplementary Note 7: Power scaling of ultra-narrow linewidth Stokes

This Supplementary note demonstrates the fibre power coupling handling and the extremely high quantum conversion to the first order Stokes.

Supplementary figure 14 shows the evolution of FS and BS with input power for a different fibre length from the one considered in the main manuscript. Here the fibre length was set to 7 m. With this length we have demonstrated a coupling with input power as high as 85.5 W. At this input power, the FS power was found to be 55 W, and BS power 3 W. With the estimated fibre coupling efficiency of 75%, we find ~97% of quantum efficiency.

Furthermore, in the main manuscript, the ultra-narrow linewidth obtained with the 20 m long fibre were limited to input powers less than 30 W. Above this input power level the generation of the second order Stokes strongly alters the SONS-GPM molecular lattice. This section is shown as a proof of concept that the lattice and hence the narrow linewidth can be obtained for higher input powers by simply shortening the fibre length and reducing the gas pressure. This will increase the input power onset at which the generation of the second-order Stokes occurs.

Supplementary figure 15 shows the linewidth traces of FS and BS generated from a 7 meter long PBG HC-PCF. The fibre is similar to the one used so far. The linewidth measurements were taken with input power up to 70 W, and the gas pressure was set to 10 bar and 20 bar. We obtained with a

pressure of 10 bar, a generated and transmitted Stokes with a power level in the range of 30-50 W and a linewidth of ~100 kHz.

Supplementary Note 8: Effective Rabi frequency definition and dependence with input power

The effective two-photon Rabi frequency Ω_{RS} acts only in the nano-traps. Consequently, its expression deviates from Ω_{12}, and its magnitude should be averaged over the wavelength, with the weight given by D. This gives the following expression:

$$\Omega_{\mathrm{RS}} = \frac{\int_z^{z+\lambda} \Omega_{12}(\mathrm{z})\mathrm{D}(\mathrm{z})\mathrm{dz}}{\int_z^{z+\lambda} \mathrm{D}(\mathrm{z})\mathrm{dz}}. \tag{14}$$

Supplementary Note 9: The phase diagram

The phase diagram is defined as a map in the (v, z) space of the value $E = \sqrt{v^2 + 2\langle U_{\mathrm{tot}}(z)\rangle/m + C}$, with $\langle U_{tot}(z)\rangle$ given in the manuscript, *C* being a constant, and $E = -\sqrt{|v^2 + 2\langle U_{\mathrm{tot}}(z)\rangle/m + C|}E$ for negative values of $v^2 + 2\langle U_{\mathrm{tot}}(z)\rangle/m + C$. The adiabatically slow motion of a molecule corresponds to motion along the line of constant *E* in the (v,*z)* space. Moreover, since *E* has units of velocity, we have chosen $C = -2\langle U_{\mathrm{tot}}(0)\rangle/m$ so that the value of *E* is equal to the velocity at *z = 0*. With such a definition, negative values of E mean localized motion.

Supplementary Note 10: The influence of the particles redistribution on the dynamics

As explained in the manuscript, the change of the gas density due to the modulated expectation value of the Hamiltonian leads to the significant, above fourfold, increase of the density in the lentils. This higher density will lead to two major effects: firstly, the population decay rate and the coherence decay rate are going to be modified. Secondly, the higher density will result in a higher collision rate of the molecules. Both of these effects, in turn, lead to the modification of the expectation value of the Hamiltonian: the former one directly as described by the formalism shown in the paper, the second one indirectly, by modifying the time molecules dwelling in any given position under the influence of the field. Therefore the distribution of the molecules over the position, velocities, and the quantum state described by the ρ_{12} and *D* should be calculated self-consistently, including the above effects. This calculation is not included in the current simplified version of theory; however, even such a simplified version gives quantitative agreement to the experimental values.

Supplementary Note 11: The moving scattering dust inside the hydrogen filled HC-PCF

Supplementary figure 16 shows a snap-shot from a video showing a scattering dust that has been trapped in the fibre guided beam. This was achieved by imaging a fibre section that was set in a spiral form so as to capture as much length as possible within a single frame. The imaging was recorded using a CCD camera in front of which is mounted an IR viewer. The speed of the scatterers was determined by recording their location within the frame in function of time, deduced from the frame number and the frame rate. The full video is available in the online supplementary materials.

Supplementary figure 17 shows the scatter's position evolution in the fibre for different input pump powers. In agreement with theory, for a given input power, the drag velocity of the scatters is constant.

Supplementary references

1. Llopis, O., Merrer, P. H., Brahimi, H., Saleh, K. & Lacroix, P. Phase noise measurement of a narrow linewidth CW laser using delay line approaches. *Opt. Lett.* **36,** 2713–2715 (2011).
2. Di Domenico, G., Schilt, S. & Thomann, P. Simple approach to the relation between laser frequency noise and laser line shape. *Appl. Opt.* **49,** 4801–4807 (2010).
